# Genome of *Mycoplasma haemofelis*, unraveling its strategies for survival and persistence

**DOI:** 10.1186/1297-9716-42-102

**Published:** 2011-09-21

**Authors:** Andrea P Santos, Ana MS Guimaraes, Naíla C do Nascimento, Phillip J SanMiguel, Samuel W Martin, Joanne B Messick

**Affiliations:** 1 Department of Comparative Pathobiology, Purdue University, 725 Harrison Street, West Lafayette, IN 47907, USA; 2 Purdue Genomics Core Facility, Purdue University, 170 S. University Street, West Lafayette, IN 47907, USA

## Abstract

*Mycoplasma haemofelis *is a mycoplasmal pathogen (hemoplasma) that attaches to the host's erythrocytes. Distributed worldwide, it has a significant impact on the health of cats causing acute disease and, despite treatment, establishing chronic infection. It might also have a role as a zoonotic agent, especially in immunocompromised patients. Whole genome sequencing and analyses of *M. haemofelis *strain Ohio2 was undertaken as a step toward understanding its survival and persistence. Metabolic pathways are reduced, relying on the host to supply many of the nutrients and metabolites needed for survival. *M. haemofelis *must import glucose for ATP generation and ribose derivates for RNA/DNA synthesis. Hypoxanthine, adenine, guanine, uracil and CMP are scavenged from the environment to support purine and pyrimidine synthesis. In addition, nicotinamide, amino acids and any vitamins needed for growth, must be acquired from its environment. The core proteome of *M. haemofelis *contains an abundance of paralogous gene families, corresponding to 70.6% of all the CDSs. This "paralog pool" is a rich source of different antigenic epitopes that can be varied to elude the host's immune system and establish chronic infection. *M. haemofelis *also appears to be capable of phase variation, which is particularly relevant to the cyclic bacteremia and persistence, characteristics of the infection in the cat. The data generated herein should be of great use for understanding the mechanisms of *M. haemofelis *infection. Further, it will provide new insights into its pathogenicity and clues needed to formulate media to support the in vitro cultivation of *M. haemofelis*.

## Introduction

*Mycoplasma haemofelis *is a hemotrophic mycoplasmal pathogen (hemoplasma) of the cat. There are two phylogenetic clusters of hemoplasmas, the haemofelis cluster and suis cluster, which appear to have descended from a common ancestor. They are most closely related to members of the pneumoniae group, albeit only peripherally. Specific characteristics distinguish the hemoplasmas from other mycoplasmas, including unique tropism for erythrocytes as well as relatively low sequence similarity of their 16S rRNA genes when compared to the closest related mucosal mycoplasma species. Nonetheless, like other mycoplasmas, *M. haemofelis *has no cell wall and is related to Gram-positive bacteria [1] from which they evolved by a reduction of their genome size [2]. Despite numerous attempts, in vitro culture of *M. haemofelis *has not been achieved.

*M. haemofelis *infection in the cat causes an acute hemolytic anemia, either directly or by initiating immune mediated destruction of red blood cells; it might also trigger the suicidal death of infected erythrocytes (eryptosis), as recently suggested for *M. suis *[3]. A wide range of clinical signs, including anemia, pyrexia, lethargy, and splenomegaly characterizes the disease, which if left untreated may result in death. *M. haemofelis *is also recognized as a pathogen in conjunction with retroviruses such as feline immunodeficiency virus (FIV), feline leukemia virus (FeLV), or other debilitating diseases [4]. Based on polymerase chain reaction (PCR) testing, 20% to as high as 40% of anemic and/or sick cats are infected with *M. haemofelis *[5-7]. Currently, there is no treatment that effectively clears the microorganisms from an infected host. Chronic infection is well recognized and even in pet cats showing no clinical signs, the prevalence of *M. haemofelis *infection may be as high as 4% [8]. While transmission of *M. haemofelis *from an infected cat to a human host has been reported [9], it appears to be a rare event and likely requires immune suppression ([10], Santos AP, unpublished observations).

Although mycoplasmas have reduced genomic sizes [1] resulting in the loss of many of their biosynthetic abilities, they retain genes important for their survival, virulence and pathogenesis. It is believed that genes encoding lipoproteins and membrane binding proteins are key factors in inducing immunity. In particular, lipoproteins are considered one of the most important pathogenic elements for mycoplasmas [11]. Phase and antigenic variation of surface proteins is thought to be pivotal to the adaptive strategies and survival of these microorganisms [12,13]. Until recently, very little was known about the membrane lipoproteins or any other proteins encoded by the genome of *M. haemofelis *[14] and related hemoplasmas [15]. Hemoplasma genes that were retained or acquired during the process of reductive evolution may be particularly valuable to understanding mycoplasmas in general and red blood cell parasitism by hemoplasmas in particular.

This study was undertaken as a step toward understanding how *M. haemofelis *has adapted to erythrocyte parasitism and persists in the blood despite the host's immune response. Furthermore, information about the biochemical pathways used by these bacteria will provide valuable clues about culture conditions needed to support their growth in vitro. Herein, we present the whole genome sequence, annotation, and bioinformatic analyses of *M. haemofelis *strain Ohio2 [16] and a comparative analysis with the recently published genome of *M. haemofelis *strain Langford 1 [17,18].

## Materials and methods

### Bacteria and DNA extraction

*M. haemofelis *was obtained from a cat experimentally infected with the Ohio2 strain; this was the second in vivo passage of blood originally obtained from an acutely ill animal. The cat was infected by intravenous injection of a thawed aliquot (1.0 mL) of infected blood from a -80°C stored stock. Prior to experimental infection, PCR testing for all feline hemoplasmas performed on 3 separate occasions was negative [19-21]. Peripheral blood was collected into EDTA tubes on 13th day post infection when 60% of the erythrocytes were infected. Infection was confirmed by microscopy and a PCR assay specific for *M. haemofelis *[19]. Microorganisms were detached and harvested from blood using a combination of filtration and ultracentrifugation procedures [22]. High-molecular-weight (HMW) *M. haemofelis *genomic DNA (g*Mhf*) was extracted using QIAGEN Genomic-tip 100/G kit (QIAGEN Inc., Valencia, CA, USA) according to the manufacturer's recommendations and purified by drop dialysis. The quality and quantity of g*Mhf *was assessed by two methods - gel electrophoresis and scanning UV spectrophotometry (NanoDrop^® ^ND-1000 UV/Vis Spectrophotometer, Thermo Fisher Scientific Inc, Wilmington, DE, USA). The cat was treated and adopted according to our animal use protocol (Purdue Animal Care and Use Committee, protocol #08-003).

### Sequencing and assembly

Whole-genome sequencing of *M. haemofelis *was performed by Purdue University's Genomics Core Facility using a GS-FLX (454) and Titanium chemistry to sequence a 3 kb paired end library. Sequences were assembled using Versions 2.3 of the Roche's GS De Novo Assembler (454 Life Sciences, Roche Applied Science, Branford, CT, USA). The assembly was examined using consed [23] and where sufficient overlap between adjacent contigs was found, they were joined into a single contig.

### Finishing and validation

Gap closure was performed by primer walking directly on the genomic DNA combined with PCR followed by bidirectional Sanger sequencing of amplicons. Gross genome validation was achieved by comparing the virtual fingerprint patterns of *M. haemofelis *genomic DNA to that of fragments derived from pulse-field gel electrophoresis (PFGE) using the restriction enzymes, *Nru*I, *Sal*I, and *Not*I, and with physical map data from an independently derived bacterial artificial chromosome (BAC) library [22]. In addition, the sequencing of 21 inserts (0.2 to 4.5 kb) from two Lambda ZAPII libraries of *M. haemofelis *[24] was accomplished and compared to the *M. haemofelis *genomic sequence.

### Optical map

Finally, a high resolution, optical map was used to provide a purely independent means of sequence validation (OpGen Technologies Inc, Madison, WI, USA) [25]. Briefly, the optical map was constructed from individual *M. haemofelis *DNA molecules cleaved with *Nco*I. In silico *Nco*I restriction map of the assembly contig of *M. haemofelis' *complete genome was constructed and compared to the *Nco*I optical map using MapSolver version 2.1.1 (OpGen Technologies Inc).

### Genome annotation

First-pass annotation was achieved using blast2GO. To confirm the results provided, the scaffold sequence of *M. haemofelis *was submitted to the annotation service, Manatee, provided by the Institute for Genome Sciences (IGS) at the University of Maryland, School of Medicine. Manual curation of each gene was achieved using the annotation tool.

### Genome analyses

To make functional assignments of predicted protein coding sequences (CDSs) several approaches were used. Assignment of the origin of replication was performed using the Ori-finder tool with parameters adjusted to specific DNA boxes for *Escherichia coli *and *Mycoplasma *species and 1 or 2 unmatched sites permitted [26]; bases in the genome were numbered starting with *dnaA *as the first gene. Comparative analyses with other bacterial genomes were performed based on genome annotations deposited in the databases at National Center for Biotechnology Information (NCBI, Bethesda, MD, USA). Paralogous gene families were assigned using BLASTclust tool at the Max-Planck Institute for Developmental Biology [27], with 30% sequence identity and 70% covered length thresholds. To identify structural features of the genome a set of software was used: Tied Mixture Hidden Markov Model, TMHMM Server v. 2.0 [28] and Dense Alignment Surface method, DAS [29] were used to predict transmembrane helices; the tandem repeats finder program was used to identify repeated sequences throughout the genome [30]; and LipoP and SignalP algorithms were used to predict lipoproteins and signal peptides, respectively [31,32]. Prediction of protein sorting signals and subcellular localization was performed using PSORTb v.3.0 [33,34]. Predictions of metabolic pathways were based on the KEGG pathway database [35] and the study performed by Yus et al. [36]. Comparative analyses of the whole genome of *M. haemofelis *strains Ohio2 and Langford 1 [GenBank: FR773153] were performed using the same methods described above. Global genomic comparisons were achieved by constructing in silico restriction maps using MapSolver version 2.1.1 (OpGen Technologies Inc).

## Results and discussion

### Sequencing and assembly

From a quarter PicoTitrePlate GS-FLX run, a total of 214 186 filter-pass sequence reads were generated with an average read length of 350 bp. This generated roughly 67 million bases of sequence after the removal of adaptor sequence. In total, 219 contigs were generated, including 55 linked by paired-end reads into a single scaffold derived from *M. haemofelis *DNA and comprising 85% of the reads. The balance of the contigs was derived from cat DNA. The draft assembly of *M. haemofelis *was 1 150 927 bp, however there were still 30 gaps and areas of questionable sequence fidelity. Due to the presence of repeated sequences, some difficulties were experienced in assembling the sequences to close one gap. Nonetheless, we were ultimately able to close all 30 gaps by PCR and primer walking, which resulted in a final sequence assembly of 1 155 937 bp.

### Validation

The optical map constructed from individual *M. haemofelis *DNA molecules cleaved with *Nco*I gave rise to 110 optical contigs with an average fragment size of 10 316 bp that were assembled into one circular consensus chromosome (Additional file 1, Figure S1). The average depth of coverage of the map was 74×, and no region in the map was less than 34× coverage. The genome size was 1 134 779 bp. In comparison, the in silico map of the *M. haemofelis *sequence generated herein consisted of 140 fragments with an average fragment size of 8 198 bp. The average length of the in silico map is 2 118 bp shorter than the average fragment length of the optical map. Since restriction fragments shorter than 500 bp cannot be detected by optical mapping, this likely explains the observed differences. Optical mapping identified an assembly error in the *M. haemofelis *sequence consisting of a large inversion in the sequence assembly, permitting the re-orientation of the data and correction of the genome sequence (Additional file 2, Figure S2). The optical map, otherwise, verified the 454 sequence assembly.

In previous studies, a bacterial artificial chromosome (BAC) library and a physical map of *M. haemofelis *was completed [22]; the size of the genome was calculated to be approximately 1 113 kb and the average GC% of coding sequences was 38.5%. Thus, our assembled sequence in this study was 3.9% larger than that predicted by restriction enzyme maps but only 1.9% larger than the estimated size by optical mapping.

### General features

**Figure 1:**
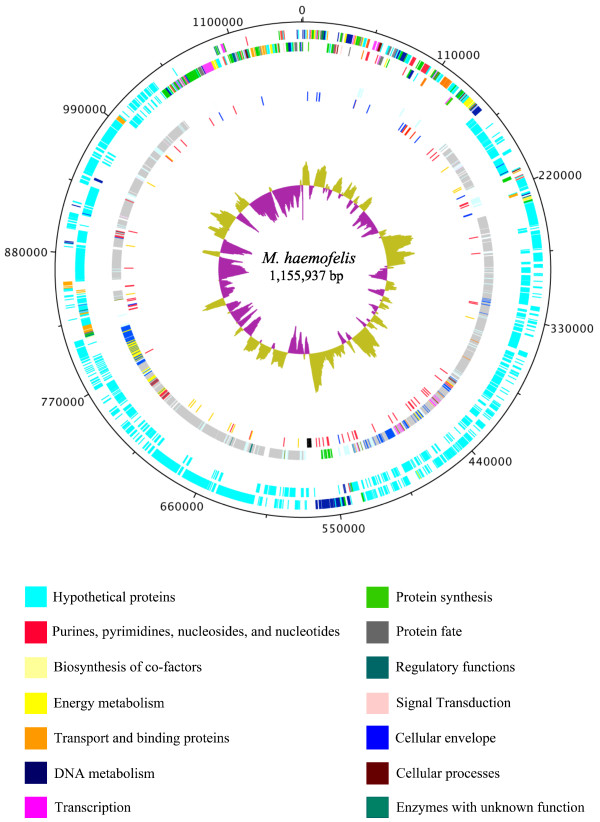
**Diagram of overall structure of *Mycoplasma haemofelis *genome**. The *dnaA *gene is at position zero. The distribution of genes is depicted on two outermost concentric circles: First concentric circle: predicted coding regions on the plus strand. Second concentric circle: predicted coding regions on the minus strand. Predicted coding regions are classified by functional categories (TIGR roles) according to the color code. Moving inwards, the third circle displays the genes that are within paralogous gene families, where same color means CDSs from the same family, except for light green representing all the families with 5 or less members. The fourth circle represents the tandem repeats (red), the 17 predicted lipoproteins (orange), rRNAs (black) and tRNAs (blue). The innermost circle represents the GC skew. The figure was generated using DNAPlotter version 1.4 from Artemis 12.0, Sanger Institute.

The general genome features of the *M. haemofelis *strain Ohio2 are shown in Figure 1 and compared with other mycoplasmas in Table 1. The genome of *M. haemofelis *consists of a single circular chromosome with a size of 1 155 937 bp and an overall guanine and cytosine percentage (GC%) of 38.8. These are typical characteristics of mycoplasmas, which have small genomes and GC% ranging from 23.8 to 40%. During the analyses of the genome of *M. haemofelis *strain Ohio2, the strain Langford 1 was reported [18]. To compare both genomes, we first constructed in silico restriction maps of each genome (Additional file 3, Figure S3). The restriction maps revealed 10 regions of the genome with significant difference in the nucleotide sequence while no inversions were observed. There is also an overall difference in size between the two genomes; the Ohio2 genome size is 1 155 937 bp, which is 8 678 bp bigger than Langford 1. Regions of gene duplications from paralogous families throughout the genome are largely responsible for the size difference.

**Table 1:** General features of *Mycoplasma haemofelis *strain Ohio2 genome compared to members of pneumoniae, hominis and mycoides phylogenetic groups of *Mycoplasmas*.

	Pneumoniae group						Hominis group				Mycoides group
**Feature**	***M. haemofelis***	***M. suis***	***M. pneumoniae***	***M. gallisepticum***	***M. genitalium***	***M. penetrans***	***M. hominis***	***M. hyopneumoniae***	***M. synoviae***	***M. pulmonis***	***M. mycoides***

Genome size (bp)	1 155 937	742 431	816 394	1 012 800	580 076	1 358 633	665 445	897 405	799 476	963 879	1 211 703
G + C content (%)	38.8	31.1	40	31	31.7	25.7	27.1	28	28	26.6	24
Total of genes	1584	883	733	817	524	1069	577	701	715	815	1053
Coding sequences	1549	844	689	763	475	1037	523	657	659	782	1017
Pseudogenes	22	4	0	14	6	0	14	11	15	0	0
Gene density (%)	94.2	89.9	88.7	91	90	88	89.8	88	91	91.4	83
Average gene length (bp)	693	783	1011	1,206	1,040	NR*^a^*	1,107	1,178	1,058	1,115	982
CDS with predicted function	299 (19.3%)	293 (34.7%)	333 (48.3%)	469 (61.46%)	323 (68%)	585 (56.4%)	345 (65.9%)	412 (62.7%)	464 (70.4%)	486 (62.1%)	581 (57.1%)
No. of tRNAs	31	32	37	32	36	29	33	30	34	29	30
No. of rRNAs											
16S	1	1	1	2	1	1	2	1	2	1	2
23S	1	1	1	2	1	1	2	1	2	1	2
5S	1	1	1	3	1	1	2	1	3	2	2
Genes in paralogous families	1103 (71.2%)	361 (42.8%)	132 (19.1%)	110 (14.4%)	25 (5.2%)	245 (23.6%)	38 (7.2%)	106 (16.1%)	121 (18.3%)	101 (12.9%)	266 (26.1%)

Data was obtained from the GenBank database using the following accession numbers: *M. haemofelis *(CP002808), *M. suis *(CP002525), *M. pneumoniae *(U00089), *M. gallisepticum *(AE015450)*, M. genitalium *(L43967)*, M. penetrans *(BA000026)*, M. hominis *(FP236530)*, M. hyopneumoniae *(AE017243)*, M. synoviae *(AE017245)*, M. pulmonis *(AL445566), *M. mycoides *(BX293980). Paralogous gene families were assigned using BLASTclust, with 30% sequence identity and 70% covered length thresholds.

*^a ^*NR, not reported.

A total of 1549 CDSs have been computationally and manually predicted. Putative biological function was assigned to 299 CDSs (19.3%), whereas 80.25% were hypothetical proteins showing no significant identity in the databases; there were 0.45% conserved hypothetical proteins (Additional file 4, Table S1). These results indicate that *M. haemofelis *genome likely encodes a large number of unique proteins. This is not surprising considering that, unlike other mycoplasmas, *M. haemofelis *has a tropism for red blood cells of the host which probably demands a different set of genes to adapt to the blood environment. Predicted CDSs are summarized by role in Table 2. Besides the differences in the genome size, all the CDSs with known function are conserved between the strains Ohio2 and Langford 1 of *M. haemofelis*. The 2 strains were manually compared, gene by gene, and only a few genes with known functions had different annotations. However, the CDSs coding hypothetical proteins varied in size and numbers; this was mainly due to gene duplications in one or the other genome (Additional file 5, Table S2).

**Table 2:** Protein coding sequences of *Mycoplasma haemofelis *strain Ohio2 genome classified by role category (TIGR roles).

Category	Number	%
Purines, pyrimidines, nucleosides, and nucleotides	29	1.85%
Fatty acid and Phospholipid metabolism	6	0.38%
Biosynthesis of co-factors, prosthetic groups, and carriers	7	0.45%
Energy metabolism	22	1.41%
Transport and binding proteins	32	2.04%
DNA metabolism	52	3.32%
Transcription	18	1.15%
Protein synthesis	97	6.20%
Protein fate	19	1.21%
Regulatory functions	3	0.19%
Signal Transduction	2	0.13%
Surface structures	7	0.45%
Cellular processes	10	0.64%
Unknown functions	8	0.51%
Conserved hypothetical proteins	7	0.45%
Hypothetical proteins	1246	79.62%

**Total**	1565*^a^*	100.00%

*^a ^*Some of the CDSs have more than one role.

The average gene length for *M. haemofelis *is shorter than other mycoplasmas but comparable with that of *M. suis *[37] (Table 1). This is a result of the presence of a large number of paralogous genes with sequences shorter than the average length; in the largest gene family the average size is 634 bp, ranging from 384 to 882 bp, while the average size of genes that are not in paralog families is 855 bp. For most bacteria, the number of paralogous genes also correlates to its genome size. However, the *Mycoplasmas *represent an exception to this tendency, having small genomes with a larger than expected percentage of paralogs [38]. Remarkably, the genome of *M. haemofelis *has the largest percentage of paralogous genes of any fully sequenced bacteria to date. This is due to the expansion of a few gene families with the largest gene family having 800 members. The presence of an extensive network of paralogs in the genome of *M. haemofelis *suggests a mechanism to support its survival as an extracellular red blood cell-associated pathogen that is continually bombarded by the host immune system.

### Replication, transcription and translation

We have assigned the origin of replication (*ori*C) based on homologous gene searches to other mycoplasma genomes and GC-skew graph predictions [39]. A conserved gene order for *rpmH*, *dnaA *and *dnaN *was observed in *M. haemofelis *genome and typically, *ori*C is located near these genes. The analysis of the GC skewing (Figure 1) showed a significant inversion near the *dnaA *gene, providing additional evidence that the origin of replication was properly located. We also attempted to identify dnaA-box motifs, which typically are found within the intergenic regions around *dnaA *gene. The Ori-finder tool did not predicted dnaA-box motifs when allowing 1 unmatched site nor when using mycoplasma or *Escherichia coli *specific dnaA-boxes. When a more open search was used (2 unmatched sites), dnaA-box motifs were predicted in several areas of the genome. Thus, we found no convincing dnaA-box motifs around the *dnaA *gene by this method. *M. suis *posseses a intergenic region of 134 bp between genes *rpmH *and *dnaA*, with only 18% of GC content, and the presence of 3 dnaA-box motifs within this area [37] leading to the prediction of the *oriC *upstream the *dnaA *gene. The origin of replication was inferred to be upstream of the *dnaA *gene in *M. haemofelis *strain Langford 1 [18]. However, this region has only 5 bp of intergenic space between *rpmH *and *dnaA *genes in both strains. Thus, we manually searched the 28 bp region between *dnaA *and *dnaN *genes; this intergenic space is exclusively composed of AT. Within this area, we identified 3 dnaA-box motifs with the consensus sequence pattern T(T/A)(T/A)A(T/A)AA. Thus, as with other bacteria, including *Mollicutes*, the origin of replication of *M. haemofelis *was mapped to an intergenic region downstream of the *dnaA *gene. Nevertheless, experimental validation is needed to conclusively locate the *ori*C of *M. haemofelis*.

Only few transcriptional factors are described in *Mycoplasma *spp. [40]. We identified 13 putative genes involved in transcription, including 3 transcriptional antitermination factors (NusA, NusB and NusG), the transcriptional elongation factor GregA, and the heat inducible transcriptional repressor HrcA. Like other mycoplasmas, *M. haemofelis *has the transcription initiation factor, the sigma factor 70 (RpoD). We also identified CDSs for the subunits of the RNA polymerase RpoA, RpoB, RpoC, and a member of the extracytoplasmic function (ECF) subfamily RpoE. The latter responds to signals from the external environment, presence of misfolded proteins and heat/oxidative stress in other bacteria [41-44] and presumably performs similar functions for *M. haemofelis*.

Several components of the translational system were identified, including the 5S, 23S and 16S ribosomal RNA genes, which are present as single copies located within the same operon, and 48 ribosomal proteins. We also identified 31 transfer RNAs (tRNAs) covering all 20 amino acids and the tRNA-SeC for the translation of the amino acid selenocysteine. The tRNAs and rRNAs are also conserved between the 2 strains of *M. haemofelis*, however 2 tRNAs are annotated differently in Langford 1 strain: HF1_t18, annotated as tRNA-Tpr instead of tRNA-Sec and HF1_t20, annotated as tRNA-Cys instead of tRNA-Arg (Additional file 6, Table S3). As in all other mycoplasmas sequenced to date [1], the opal stop codon (UGA) is used to encode tryptophan in *M. haemofelis*. The tRNA-SeC also uses the opal stop codon to insert selenocysteine in bacteria [45], but whether hemoplasmas use selenocysteine is still unclear. Other CDSs of the translation system are: 23 tRNA synthetases and 12 translation factors including the 3 prokaryotic initiation factors (IF-1, IF-2 and IF-3) and 5 GTP-binding proteins, including LepA, EngA and EngB. The peptide release factor 1 (RF-1), responsible for the recognition of stop codons UAA and UAG to terminate translation, was identified in *M. haemofelis *genome, while the peptide release factor 2 (RF-2) is missing. The RF-2 helps terminate the translation at UGA codons in other bacteria and is absent in all mycoplasmas sequenced to date.

### Restriction and modification (R-M systems)

The mechanisms of regulating gene expression are poorly understood in *Mycoplasmas*. The low number of transcription factors suggests that other regulation mechanisms might occur in these organisms. Mechanisms of restriction and modification are known to protect the bacteria against invading DNA or phage infection, but might also be responsible for genome rearrangements [46]. Phase variation of these mechanisms is proposed to increase the variability of proteins expressed [40], and ultimately adaptation to the host niche.

Type I restriction systems are multifunctional enzymes complexes that can catalyze both modification/methylation and restriction. The system activity is performed by a holoenzyme with 3 subunits for specificity (HsdS), modification (HsdM) and restriction (HsdR), which cleaves double stranded DNA randomly through out the genome [47]. *M. haemofelis *genome have 2 areas containing type I restriction enzymes; however, only one area contains the entire complex HsdS/HsdM/HsdR. Within this area we identified 21 CDSs for the HsdS subunit (9 are truncated), 2 CDSs for the HsdM, and 2 CDSs for the HsdR subunit. Interestingly, 19 CDSs of the HsdS subunit are within a family of paralogs and contain several inter and intragenic tandem repeats. This might indicate that the expression of these genes is regulated by phase variation. Phase-variable type I restriction enzymes were also identified in the *M. pulmonis *genome, for which an association with antigenic variation was suggested [48].

Type II restriction activity, the simpliest of the R-M systems, is performed by two distinct enzymes, a sequence-specific endonuclease and a DNA methyltransferase [40]. We identified enzymes of the type II R-M system in 2 areas of the genome of *M. haemofelis*, however only one area had the gene pair - a deoxyribonuclease, *Sau*96I-like and a C-5 cytosine-specific DNA methylase. The second location had only the DNA methylase. A *Sau*96I-like endonuclease also has been reported in the genome of *M. mycoides *subsp. *mycoides *and subsp. *capri *[GenBank: NC_005364.2 and NC_015431.1]. Type III restriction enzymes were not identified in the genome of *M. haemofelis*. In the pneumoniae clade, the Type III R-M system related sequences were also not found in the genomes of *M. genitalium*, *M. pneumoniae*, and *M. gallisepticum *[40].

### Protein homeostasis

Chaperone and protease families are highly conserved across most bacterial genomes and play important roles in stabilizing protein conformations, refolding misfolded proteins and degrading protein that may be detrimental to the cell's survival [49]. While two prominent chaperone systems (GroE and DnaK) are present in most bacteria, the members of the GroE protein homeostatic network, GroEL/GroES, are absent from *M. haemofelis *genome. Several other mycoplasmas have been reported to specifically lack the GroE chaperone system [50]. GroE system was also absent from the genome of *M. suis *[37], although the presence of GroEL protein has been reported previously [51]. The complete DnaK chaperone system was found in the genome of *M. haemofelis*, suggesting that the DnaK-DnaJ-GrpE complex may provide significant control over protein folding for this and other GroE chaperone deficient mycoplasmas. Another chaperone, the trigger factor (Tig) was also identified in the genome of *M. haemofelis*, but was absent in *M. suis*. Tig is involved in shielding nascent polypeptides on the ribosome, thereby preventing their degradation. Unlike the GroE and DnaK chaperone systems, Tig functions independently of ATP and its expression is upregulated in cold shock [52]. Thus, it seems unlikely that Tig could replace the missing functions of the GroE system in *M. haemofelis*.

Proteases also play a key role in the viability of bacterial cells, especially as it relates to degrading misfolded or aggregated proteins [53]. In the genome of *M. haemofelis*, the proteases identified included the heat-shock ATP-dependent protease Lon and a membrane anchored FtsH. The ATPases from the Clp family, HslU and HslV, on the other hand, are missing from the genome. It is likely that these proteases in *M. haemofelis *might also function to assist with the breakdown of imported peptides for protein synthesis.

It has been suggested that loss of specific proteases and the chaperone GroEL/GroES system may result in a shift toward proteolysis rather than protein folding as a means of maintaining protein homeostasis in mycoplasmas [52]. These shifts might play a contributing role to both survival and pathogenesis, leading some groups to investigate their potential as antimicrobial targets [54]. The functional consequences of these gene losses in *M. haemofelis *are unknown.

### Secretion and transport

*M. haemofelis *appears to use the general secretion (Sec) pathway [55] for translocation of newly synthesized proteins across the cytoplasmic membrane. The essential membrane receptor ATPase SecA, and members of the integral membrane complex SecG, SecY, and SecD (responsible for release of the mature peptide) are present. Whereas, like most other mycoplasmas, SecE (also part of the integral membrane complex), SecF and the non-essential chaperone SecB are missing. The absence of SecB suggests that the export is via signal recognition particle (SRP) [56]. The presence of the associated inner membrane protein translocase YidC [57] and 2 signal recognition particle proteins (FtsY and Ffh), that are responsible for delivering protein to SecA, reinforces this hypothesis. The Sec-independent twin arginine translocation (Tat) secretion pathway is an alternate route for folded proteins and co-factor-bound enzymes secretion [58] in bacteria. Only one member of the Tat pathway, TatD, was identified in the *M. haemofelis *genome, while essential components of the translocase, TatA, TatB, and TatC, which in conjunction with TatE form a translocation pore, are all missing. Since the TatD protein does not appear to be required for operation of the Tat transport system, we speculate that the TatD probably performs a different function, unrelated to protein transport in *M. haemofelis*, and a functional Tat secrection pathway is absent. Perhaps TatD in *M. haemofelis *can function as a magnesium-dependent DNase as previously described, thus suggesting a role for this protein in DNA metabolism [59].

Transporter systems are conserved among bacteria and are responsible for the transport of a wide range of molecules across the membrane, including the import of nutrients and export of toxins. Therefore, they are crucial for bacterial metabolism and play a role in virulence [60]. *Mycoplasmas *are known to have fewer transporters than other bacteria, suggesting that their transporters may have broader substrate specificity [36]. *M. haemofelis *also dedicates only 2.2% (34/1549) of its CDSs, configured as operons, to transport and binding proteins. As with most other mycoplasmas, ABC transporters in *M. haemofelis *represent more than 50% of all membrane transport proteins [61]; they are 70.6% (24/34) for *M. haemofelis*. The phosphotransferase transport system (PTS) is also represented in the genome of *M. haemofelis*, suggesting that sugar is acquired from the environment and translocated across the cell membrane [62] for use in energy metabolism.

### Energy metabolism

*M. haemofelis *uses the Embden-Meyerhof-Parnas (EMP) pathway (glycolysis) for energy metabolism (Additional file 7, Figure S4a). The presence of CDSs in *M. haemofelis *for the PTS and enzymes responsible for the conversion of glucose into pyruvate molecules is consistent with an organism that undergoes glycolysis. Interestingly, a non-phosphorylating NADPH-dependent glyceraldehyde-3-phosphate dehydrogenase (GAPN), responsible for a shunt in the glycolysis pathway by converting glyceraldehyde-3-phosphate into 3-phosphoglycerate, is present. This enzyme reduces NADP to NADPH and has been described in some mycoplasmas and a few other bacterial species as a means of oxidative damage resistance and NADPH regeneration [63-65]. As described in other *Mollicutes*, the F0F1 ATP synthase complex is present, which can also generate ATP.

The pyruvate metabolism of *M. haemofelis *does not appear to be complete. Orthologs to the pyruvate dehydrogenase complex and acetate kinase are absent. Enzymes of the coenzyme A metabolism are also not present, which supports the lack of a pyruvate dehydrogenase complex.

### Nicotinate/Nicotinamide metabolism

Molecules of NAD^+ ^and NADP^+ ^are end-products of the niacin (nicotinate and nicotinamide) metabolism (Additional file 7, Figure S4b). We speculate that due to the absence of two key enzymes from the nicotinate metabolism (nicotinate phosphorybosyltransferase and NAD^+ ^synthase), *M. haemofelis *is more likely to use nicotinamide as a precursor for NAD^+ ^and NADP^+^. This latter pathway would include the use of the purine nucleoside phosphorylase, which also participates in the purine metabolism, and the use of a yet to be identified ribosylnicotinamide kinase, followed by the nicotinate-nucleotide adenylytransferase. Metabolic pathway comparisons show that *M. suis *has the same enzymes to produce NAD^+^, but a NAD^+ ^kinase, present in *M. haemofelis*, is absent in the pig pathogen [37]. This enzyme is responsible for the interconversion of NAD^+ ^and NADP^+^, which plays a critical role in maintaining the NADH/NADPH pool balance inside the bacterial cell. Thus, nicotinamide is likely to be the only requirement for NAD^+ ^and NADP^+ ^production in *M. haemofelis*, whereas in *M. suis*, NADP^+ ^is possibly also needed.

### Vitamin metabolism

Although the nine vitamins (nicotinate spermine, thiamin, pyridoxal, thioctic acid, riboflavin, choline, folic acid and coenzyme A/panthothenate) are necessary for optimal growth, none of these are synthesized by mycoplasmas [36]. The only enzyme related to vitamin metabolism present in *M. haemofelis *is the serine hydroxymethyltransferase of the folate metabolism, which was not found in *M. suis*. Consequently, *M. haemofelis *may interconvert L-serine to glycine, but to do so it needs folate derivatives that are likely acquired from its environment. Thus, the author's speculate that folate derivatives and other vitamins must be imported from the bacteria's environment to support its growth and survival.

Like *M. suis *[37], *M. haemofelis *possesses spermidine/putrescine transport system consisting of a membrane associated ATPase (PotA), two transmembrane proteins (PotB and PotC), and a periplasmic substrate-binding protein (PotD). All of these proteins are necessary for the uptake of spermidine and putrescine, which play a vital role in the DNA and RNA metabolism and regulation of RNA and protein synthesis [66]. However, the enzymes necessary for the synthesis of spermidine/putrescine are missing, suggesting that *M. haemofelis *imports these polyamines from the environment. Similarly, *M. haemofelis *possesses a transporter for cobalamine, suggesting this vitamin is also essential for its survival.

### The pentose phosphate pathway

As observed in phytoplasmas [67] and in *M. suis *[37], the pentose phosphate pathway is absent in *M. haemofelis*. This mycoplasma is likely to use other means of NADPH regeneration and ribose production, including acquiring it from the environment. Since a ribokinase was not found in the genome of *M. haemofelis*, it is more likely that this organism imports deoxyribose or ribose/deoxyribose 5' phosphate than ribose to produce phosphoribosyl pyrophosphate (PRPP). The latter enzyme plays a critical role in purine/pyrimidine nucleotides synthesis (Additional file 7, Figure S4c and d).

### Purine metabolism

Several studies have demonstrated that *Mollicutes *lack the ability to synthesize de novo purine and pyrimidine nucleotides resulting in the development of unique mechanisms used to import precursors from their environment [68-70]. Of particular interest, CDSs for enzymes of purine nucleotide production from hypoxanthine are present in the *M. haemofelis *genome (Additional file 7, Figure S4c). This pathway may represent an adaptation to the blood environment; hypoxanthine is a metabolite secreted as an end product from red blood cell nucleotide metabolism and is required for the in vitro growth of *Plasmodium *spp, another red blood cell parasite [71,72]. *M. haemofelis *posseses 2 copies of the enzyme hypoxanthine phosphoribosyltransferase that not only converts hypoxanthine into inosine 5' monophosphate, but can also convert guanine into guanine 5' monophosphate (GMP). However, hypoxanthine by itself is able to serve as a precursor for purine nucleotides. *M. haemofelis *is also capable of producing GTP and dGTP from guanosine and ATP and dATP from adenine. The latter reaction is catalyzed by the enzyme adenosine kinase, which is unique to *M. haemofelis *among the other mycoplasmas sequenced to date.

Interestingly, guanine and adenine can also possibly serve as unique precursors of all purine nucleotides if the first 2 reactions of each pathway have reversibility. In this case, if guanine is used, glutamate must be available. Whereas, if adenine is used, fumarate must be available: fumarate can be acquired from the L-aspartate metabolism. These data suggests that *M. haemofelis *might import hypoxanthine, adenine and/or guanine to be used as precursors for the purine nucleotides ATP/GTP (RNA) and dATP/dGTP (DNA).

### Pyrimidines metabolism

Like most mycoplasmas, *M. haemofelis *lacks orotate-related genes needed for the synthesis of pyrimidines [73]. Uracil, however, can serve as precursor for uracil and cytosine nucleotides for RNA production in *M. haemofelis *(Additional file 7, Figure S4d). Regarding DNA production, the absence of the enzyme thymidine phosphorylase suggests that thymidine is likely the precursor imported for dTTP production. Despite the similarities to the *M. suis *pyrimidine metabolism, *M. haemofelis *has a cytidylate kinase, which can convert cytidine 5' monophosphate (CMP) to cytidine 5' diphosphate (CDP). The absence of this enzyme in *M. suis *[37] led to the speculation of the use of a phosphofructokinase to generate CDP from cytidine 5' triphosphate (CTP) [74], which implies that uracil can be used to produce cytosine nucleotides. In *M. haemofelis*, dCDP is likely produced from cytosine derivatives, not from uracil. However, enzymes responsible for the generation of CMP from cytidine or cytosine were not found. It is thus unknown which cytosine derivative (cytosine, cytidine or CMP) must be imported for dCTP production.

Thus, the addition of hypoxanthine, adenine, guanine, uracil, cytosine derivatives, and thymine/thymidine could theoretically help to sustain *M. haemofelis *DNA and RNA production in future attempts to cultivate this organism in vitro.

### Lipid metabolism

Phospholipids, glycolipids and sterols, are the three major lipid constituents of cell membranes. Mycoplasmas are thought to be completely incapable of fatty acid biosynthesis from acetyl-CoA, probably due to the loss of genetic material [75]. Although some of the enzymes are missing, *M. haemofelis *is likely to synthesize phospholipids from glycerol (Additional file 7, Figure S4e). The presence of CDS for glycerol kinase indicates that *M. haemofelis *produces glycerol-3-phosphate, an important precursor for phosphatidate, the simplest phospholipid to be incorporated into biological membranes. There are some gaps in this pathway, which include the enzymes: glycerol-3-phosphate O-acyltransferase, 1-acyl-sn-glycerol-3-phosphate acyltransferase. These enzymes perform acylation of glycerol compounds using the acyl-carrier protein (ACP) as a donor. This protein, as well as the coenzyme A (CoA)-biosynthesis pathway that acylates it, are absent in *M. haemofelis*. Likewise, the ACP is missing in *M. hyopneumoniae*, but other enzymes of the CoA metabolism are present. It is likely that *M. haemofelis *uses another mechanism of acyl transfer. From phosphatidate to cardiolipin formation, there is only one enzyme missing, the phosphatidyl glycerophosphatase. The role of this enzyme may be replaced by the enzyme cardiolipin synthase, which can convert cytidine 5' diphosphate diacylglycerol directly to cardiolipin in the presence of phosphatidylglycerol. Thus, we speculate that phosphatidylglycerol is acquired from the blood or another enzyme might produce this phospholipid.

Given the presence of choline kinase and choline-phosphate cytidylyltransferase, phosphatidylcholine is also probably synthesized. In addition, we predict that *M. haemofelis*, like other mycoplasmas, require the addition of exogenous sterols for their in vitro growth.

### Amino acid metabolism

Like other mycoplasmas, *M. haemofelis *has lost the genes required for amino acid metabolism, and is unable to synthesize any of the amino acids. Surprisingly, only one amino acid permease ABC transporter was identified in the genome of *M. haemofelis*. Since amino acids are essential for optimal growth of mycoplasmas [36], it is likely that ABC transporters in the genome of *M. haemofelis *having unknown substrate specificity or other transporters yet to be identified, may be responsible for this uptake.

### Potential virulence factors

Bacteria have evolved several virulence factors that enable them to establish an infection, which include the production of cytolysins, toxins, and invasins. *Mycoplasma *genomic repertoires, as a general feature, appear to contain few of these genes [76]. Nonetheless, we identified 2 CDSs of primary virulence genes in the genome of *M. haemofelis*. The first of these, endopeptidase o-sialoglycoprotein, was also found in the *M. suis *genome [37]. This enzyme might be directly involved in erythrocyte lysis by cleavage of glycoproteins such as glycophorin A, which is an abundant component of the erythrocyte membrane. Secondly, we confirmed the presence of a CDS for the superoxide dismutase (SOD) gene that was previously described in a sequencing survey of *M. haemofelis *[22]. The gene encoding this enzyme has not been found in genomes of other mycoplasmas despite finding evidence of its activity [77] and is absent from the genome of *M. suis*, the only other fully sequenced hemoplasma [37]. This might suggest a role for SOD in detoxifying reactive oxygen species, thus protecting *M. haemofelis *from the onslaught of oxidant damage that it encounters in its red blood cell niche. The contribution of this enzyme to the virulence of *M. haemofelis*, particularly its role as a primary pathogen, is an intriguing question for future investigations. *M. suis*, on the other hand, is not a primary pathogen and requires splenectomy of the pig for development of acute disease.

Lipid-associated membrane proteins are well described in mycoplasmas and are the preferential target for the host's immune response [78]. A total of 17 putative lipoproteins were identified in the genome of *M. haemofelis *(Figure 1). In addition, some putative surface lipoproteins were recently identified by immune screening of an expression library of *M. haemofelis *[24].

To allow for the presence of diverse subpopulations that can quickly respond to changing environmental conditions, a common virulence feature in the genome of several *Mycoplasma *species is the presence of strategically located tandem repeats [79]. Likewise, we identified 61 variable number tandem repeats (VNTR) in the genome of *M. haemofelis *(Additional file 8, Table S4) whereas *M. suis *had 33 (Guimaraes AMS, unpublished observations). Most of the VNTRs were within intergenic regions of hypothetical proteins (Figure 1). Additionally, VNTRs flanking the CDS for RNA polymerase sigma factor RpoD were identified. This feature was also found in the genome of *M. hyopneumoniae *[50]. Several VNTR's were identified within the Type I restriction system operon, as described above. Since the gain or loss of nucleotides in the promoter region would act as an on/off switch for promoter activity, it is speculated that these genes might code for phase variable surface proteins in *M. haemofelis*. Thus, as with other mycoplasmas, the strategic placement of these repetitive sequences may be related to size and phase variation [80]. While these repetitive sequences are somewhat scattered throughout the genome, there are distinct clusters (Figure 1). Palindromic structures, overlapping of repeats and degenerate repeats were found in the genome of *M. haemofelis *as well as that of *M. suis *(Guimaraes AMS, unpublished observations); these structures also have been shown to be related to virulence and bacterial gene regulation [79]. The role that these structures might play in helping *M. haemofelis *evade host defenses, adapt to its microenvironment, and establish chronic infection is an area of current investigation.

Despite their relatively small size, a high proportion of the genome in many mycoplasmas is dedicated to paralogous genes [73]. There is strong evidence to support a role for these genes in the development of antigenic diversity and the ability of the organism to avoid the host's immune response [80]. Search results using BLASTclust showed that 1093 out of the 1549 (70.6%) predicted proteins formed 46 paralogous gene families, ranging from 2 to 800 CDSs per family (Additional file 9, Table S5). Although the genome of *M. suis *is much smaller, this closely related hemoplasma relative of *M. haemofelis *still devotes 42.8% of its genome to paralogs [37]. Using the same analysis conditions for identification of paralogs, the genomes of other mycoplasmas were analyzed and compared to *M. haemofelis *(Table 1). *M. haemofelis *has more genes organized in paralogous families than all the mycoplasmas sequenced to date. The paralogs in the *M. haemofelis *genome are mostly hypothetical proteins, including the 3 largest families. Most of them have members with internal alpha helices or signal peptides, except for one family (family 3) with CDSs predicted to be cytoplasmic (Table 3). Interestingly, CDSs of family 5 have conserved motifs matching with the *Staphylococcus aureus *fibrinogen-binding protein A (clumping factor), an adhesin responsible for its attachment to fibrinogen/fibrin [81]. Thus, this family of membrane-associated putative proteins might act as adhesins in *M. haemofelis*.

**Table 3:** Analyses of the largest paralogous gene families (*n *> 5) in *Mycoplasma haemofelis *strain Ohio2 genome.

**Family**	**Number of CDSs**	**Predicted function**	**Prediction*^a^***		**Subcellular Localization*^a^***			
				
			**Internal Helix (> 1)**	**Non-Cytoplasmic (signal peptide)**	**Cytoplasmic**	**Cytoplasmic Membrane**	**Extracellular**	**Unknown**
	
1	800	Hypothetical proteins	673	136	30	15	70	685
2	99	Hypothetical proteins	94	43	8	8	9	74
3	26	Hypothetical proteins	22	0	23	0	0	3
4	18	Type I restriction enzymes	0	0	15	0	0	3
5	17	Hypothetical proteins	2	10	1	13	0	3
6	11	Hypothetical proteins	8	1	0	2	0	9
7	10	Hypothetical proteins	10	4	2	0	0	8
8	8	Hypothetical proteins	8	1	3	0	2	3
9	7	Hypothetical proteins	1	3	2	0	0	5
10	6	Hypothetical proteins	4	2	3	1	0	2
11	5	Hypothetical proteins	5	2	0	0	0	5

*^a ^*PSORTb version 3 software.

Using the same BLASTclust parameters, the total number of genes of in *M. haemofelis *strain Langford 1 within paralog families is 1042 (67.5% of CDSs). The paralogous gene families are conserved between the 2 strains of *M. haemofelis*, except that strain Langford 1 has 723 CDSs in its largest family compared to 800 CDSs in Ohio2 (family 1). Further, strain Langford 1 has 2 additional families of 25 and 17 hypothetical proteins; in strain Ohio2, these proteins are within family 1.

Strikingly, *M. haemofelis *devotes a high percentage of its genome to paralogous gene families, a feature suggesting that evasion of the immune response is a high priority for this pathogen. While these findings provide evidence to support the presence of antigenic variation by *M. haemofelis*, experimental studies are still needed to understand the role and/or function of these structures.

Only one gene related to antimicrobial resistance, a ribosomal RNA adenine dimethylase family protein (MHF_1613), was identified in the genome of *M. haemofelis*. The product of this gene is a methylase, responsible for modification of the 16S rRNA, optimizing ribosome function and consequently translation. Lack of methylation by this enzyme modifies the ribosomal binding site for the aminoglycoside antimicrobial kasugamycin, leading to resistance [82]. Whether or not this enzyme plays a role in antimicrobial resistance for *M. haemofelis *must be experimentally confirmed.

### Bacterium-host interactions

*M. haemofelis*, unlike most other hemoplasmas, is capable of acting as a primary pathogen and can cause acute disease in immunocompetent hosts. Our laboratory recently proposed a new model based on genomic findings for the acute disease caused by *M. suis *in pigs [37]. We believe that a nutrient competition and scavenging mechanism reduces the production of energy by the erythrocyte leading to oxidative stress and shortened life span of the cells, which are prematurely removed from the circulation, contributing to the development of anemia. The depletion of energy and oxidative stress may also be a trigger to eryptosis, as recently proposed for *M. suis *infection in pigs [3]. Based on the genomic information herein, it is likely that *M. haemofelis *exerts similar damage to the cat's red blood cells. Additionally, it is possible that the increased virulence of *M. haemofelis *(its ability to act as a primary pathogen) is due to decreased susceptibility to reactive oxygen species generated by the host or by the bacteria itself. The superoxide dismutase activity, thus, contributes to survival of *M. haemofelis*, and indirectly to its virulence. Since the end product of SOD activity is H_2_O_2_, the possibility that the enzyme is contributing to the virulence of *M. haemofelis *cannot be discounted [18].

The disease presentation and genomic evidence suggest that the pathogenesis of *M. haemofelis *is also linked to its antigenically dynamic cell surface. Thus, the ability of this microorganism to change its surface features might explain the cyclic bacteremic episodes that are characteristic of *M. haemofelis *infection [83-85] and persistence of the organism despite the host's immune response and/or antimicrobial treatment [86]. It is of particular interest that cattle infected with *Anaplasma marginale*, another red blood cell bacteria, have a persistent infection characterized by cyclic bacteremic bouts. Each of these bouts appears to be associated with the emergence of new antigenic variants, which are derived from paralogous gene family recombination [87]. Given that the genome of *M. haemofelis *is replete with paralogs as well as tandem repeats, we speculate that either phase and/or antigenic variation may be involved in the development of cyclic episodes and persistence of infection. The host presumably clears some of the microorganisms (nadir of the cycle), but *M. haemofelis *expressing variant antigens, not yet identified by the immune system, persist and multiply (peak of the cycle). The failure of several groups [88,89] to find microorganisms sequestered in tissues, other than on erythrocytes in the blood, adds further support to this hypothesis.

Although antigenic variation might explain the initial fluctuations in bacteremia, it does not explain why the cycling progressively declines and may disappear with time. It is possible that the host establishes an antibody response against non-variable antigens that helps to maintain a low bacterial load or that antibodies formed against variable antigens cross-react to some extent and are able to partially control the bouts of bacteremia. Another possible explanation is that the prolonged high loads of bacteremia during infection of *M. haemofelis *result in a functional dysregulation of specific CD4^+ ^T cell response. Thus, as reported for *A. marginale*, the failure to establish a strong memory T cell response may contribute to bacterial persistence [90]. Further studies are underway to identify variable antigenic families, as well as the possible mechanisms of immune evasion by *M. haemofelis *infection in cats.

In summary, the pathogenicity of *M. haemofelis *appears to be closely linked to intrinsic metabolic or catabolic pathway functions and to its dynamic outer surface. Virulence attributes of this bacterium allow it to evade the immune system of the host, adhere to the red blood cells and rapidly multiply, disseminate and persist if the host survives acute infection. It is possible that establishment of chronic infection facilitates transmission of the bacteria. The data generated in this study should be of great value for future experiments aimed at understanding the mechanisms this organism employs in colonization of red blood cells, development of disease and persistent infection. Further, it will provide new insights into the regulation of virulence factors and provide clues needed to formulate media to support the in vitro cultivation of *M. haemofelis*.

### GenBank accession number

The sequence was submitted to the GenBank database under the accession number CP002808.

## Competing interests

The authors declare that they have no competing interests.

## Authors' contributions

Conceived and designed experiments: APS, AMSG, JBM. Performed experiments: APS, AMSG, SWM, JBM. Analyzed the data: APS, AMSG, NCN, PJS, JBM. Wrote and reviewed the paper: APS, AMSG, NCN, PJS, JBM. All authors read and approved the final manuscript.

## Supplementary Material

Additional file 1 **Figure S1: Optical map of *M. haemofelis *strain Ohio2 cleaved with *Nco*I (OpGen, Madison, WI, USA)**. The outermost color circle is the consensus map and is built from the underlying maps represented as arcs. Congruent restriction fragments shown in the consensus map are denoted by a common color; the color-ordering scheme is random to provide contrast. Click here for file

Additional file 2 **Figure S2: Validation of the *M. haemofelis *strain Ohio2 sequence assembly by optical map comparison**. Vertical lines represent the restriction site. Regions with similarities are illustrated in blue, regions with differences are illustrated in white. Click here for file

Additional file 3 **Figure S3: Comparative analysis of the whole genomes of *M. haemofelis *strains Ohio2 and Langford 1 by in silico restriction maps**. Vertical lines represent the restriction sites. Regions with similarities are illustrated in blue, regions with differences are illustrated in white. (a) Restricted with *Nco*I (6 cutter), and (b) Restricted with *Eco*RI (4 cutter). Click here for file

Additional file 4 **Table S1: Protein coding sequences of *M. haemofelis *strain Ohio2 which are currently assigned to TIGR microbial role categories, and sorted by role category**. This list was generated using the Manatee annotation tool, Institute for Genome Sciences, School of Medicine, University of Maryland. Click here for file

Additional file 5 **Table S2: Comparison of the coding sequences of *M. haemofelis *strains Ohio2 and Langford 1 genomes**. BLASTp was used for protein comparisons, when no match was found, BLASTn was also use to avoid misinterpretation of annotation differences. NA = not annotated or annotated differently; X = CDSs not found in the genome; * Pseudogenes. Gray lines represent the regions with differences correspondent to Figure S3 (b). Click here for file

Additional file 6 **Table S3: Comparison of the non-coding sequences of *M. haemofelis *strains Ohio2 and Langford 1 genomes**. *Annotated differently in the *M. haemofelis *strain Langford 1 genome. Click here for file

Additional file 7 **Figure S4: Predicted metabolic pathways of *M. haemofelis *strain Ohio2**. Gray boxes represent substrates or products. Gray ellipses are metabolites predicted to be imported from the extracellular environment. White boxes represent enzymes with orthologs in the genome of *M. haemofelis*. Dashed white boxes represent enzymes with no orthologs in the genome of *M. haemofelis*. (a) Glycolysis, (b) Nicotinate/Nicotinamide metabolism, (c) Purine metabolism, (d) Pyrimidine metabolism, (e) Lipid metabolism. Pathway predictions were based on KEGG pathway database [35] and the study performed by Yus et al. [36]. Click here for file

Additional file 8 **Table S4: Tandem repeats in *M. haemofelis *strain Ohio2 genome**. This list was generated using the Tandem Repeats Finder Program. Click here for file

Additional file 9 **Table S5: List of GenBank accession numbers of CDSs of *M. haemofelis *strain Ohio2 genome distributed in paralog families**. This list was generated using the BLASTclust software. Click here for file
