# Supplementary material for: Genome of Mycoplasma haemofelis, unraveling its strategies for survival and persistence
Source: Vet Res. 2011 Sep 21;42(1):102. doi: 10.1186/1297-9716-42-102 (PMC3196708; doi:10.1186/1297-9716-42-102)
Supplement: Additional file 1 — Figure S1: Optical map of M. haemofelis strain Ohio2 cleaved with NcoI (OpGen, Madison, WI, USA). The outermost color circle is the consensus map and is built from the underlying maps represented as arcs. Congruent restriction fragments shown in the consensus map are denoted by a common color; the color-ordering scheme is random to provide contrast. [file 1297-9716-42-102-S1.PDF]

Optical Map of M. haemofelis - NcoI

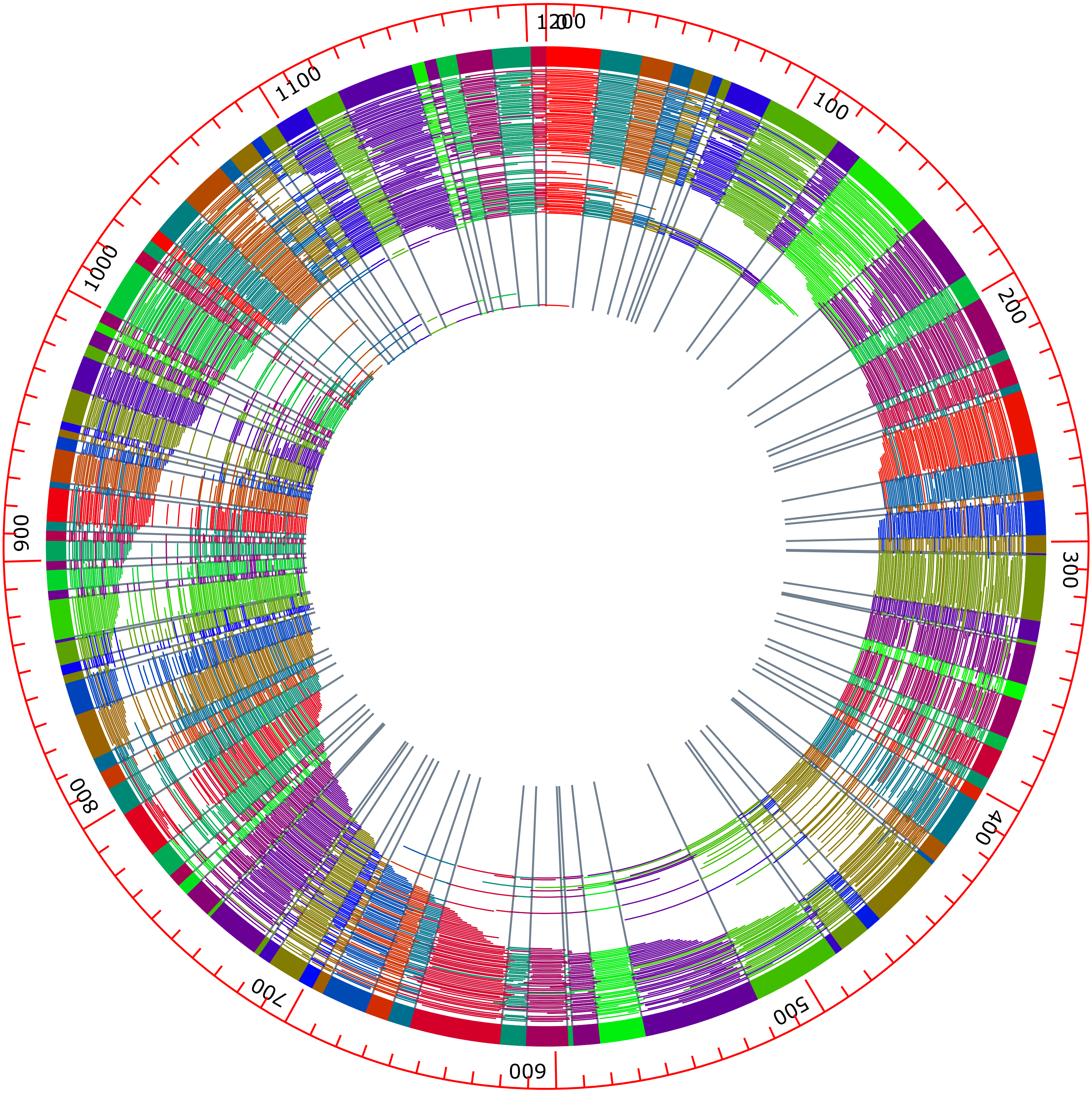

Optical map of *Mycoplasma haemofelis* Ohio2 cleaved with *Nco* I (OpGen, Madison, WI).

The outermost color circle is the consensus map and is built from the underlying maps represented as arcs. Congruent restriction fragments shown in the consensus map are denoted by a common color; the color-ordering scheme is random to provide contrast.

Restriction map of *M. haemofelis* Ohio2 *Nco* I

The columns below show statistics for every fragment in the final physical map. The 'Consensus' is the final fragment size and the 'Standard Deviation' is the standar deviation of the fragments used to determine the consensus fragment.

Total length (kb): 1207

Total fragments: 110

Molecule maps: 409

Total coverage: 74

Fragment	Size (kb)	Standard Deviation (kb)
0	21.445	0.205
1	15.963	0.181
2	12.477	0.186
3	8.207	0.157
4	7.620	0.183
5	3.776	0.138
6	3.681	0.120
7	16.530	0.226
8	30.338	0.280
9	10.389	0.192
10	34.236	0.339
11	26.903	0.242

12	10.589	0.159
13	21.791	0.254
14	4.000	0.102
15	9.954	0.207
16	3.076	0.083
17	24.592	0.223
18	14.789	0.217
19	3.524	0.090
20	13.623	0.158
21	7.050	0.276
22	0.684	0.078
23	25.576	0.233
24	8.062	0.212
25	1.395	0.057
26	15.630	0.187
27	6.186	0.119
28	15.505	0.201
29	5.497	0.118
30	11.562	0.181
31	4.935	0.123
32	5.027	0.117
33	20.647	0.243
34	7.499	0.258
35	1.647	0.096
36	28.927	0.349

37	6.123	0.198
38	12.445	0.328
39	2.944	0.155
40	34.751	0.459
41	45.433	0.400
42	17.774	0.272
43	10.291	0.317
44	1.976	0.098
45	16.375	0.247
46	10.018	0.188
47	35.380	0.304
48	8.781	0.152
49	9.100	0.162
50	17.959	0.234
51	4.553	0.111
52	5.916	0.126
53	13.072	0.204
54	5.087	0.162
55	2.114	0.071
56	21.747	0.274
57	1.437	0.070
58	10.975	0.194
59	4.634	0.100
60	5.060	0.105
61	10.519	0.151

62	18.914	0.174
63	11.399	0.137
64	7.163	0.108
65	5.773	0.096
66	18.170	0.176
67	12.806	0.218
68	3.271	0.089
69	3.902	0.084
70	8.767	0.240
71	1.129	0.067
72	15.643	0.182
73	3.528	0.082
74	8.019	0.147
75	3.448	0.088
76	7.901	0.131
77	3.873	0.096
78	3.615	0.089
79	13.110	0.202
80	2.322	0.065
81	12.779	0.178
82	4.850	0.136
83	2.881	0.101
84	3.099	0.077
85	13.064	0.145
86	13.353	0.173

87	4.775	0.110
88	6.033	0.142
89	3.564	0.108
90	4.888	0.113
91	21.965	0.246
92	5.297	0.119
93	4.961	0.103
94	4.888	0.114
95	15.596	0.194
96	18.137	0.250
97	5.220	0.118
98	10.195	0.165
99	4.389	0.108
100	7.258	0.139
101	13.479	0.165
102	13.572	0.199
103	30.058	0.338
104	5.068	0.132
105	4.591	0.105
106	7.884	0.125
107	13.884	0.166
108	15.027	0.180
109	5.880	0.113
