# Supplementary figures and images for: Genome of Mycoplasma haemofelis, unraveling its strategies for survival and persistence
Source: Vet Res. 2011 Sep 21;42(1):102. doi: 10.1186/1297-9716-42-102 (PMC3196708; doi:10.1186/1297-9716-42-102)

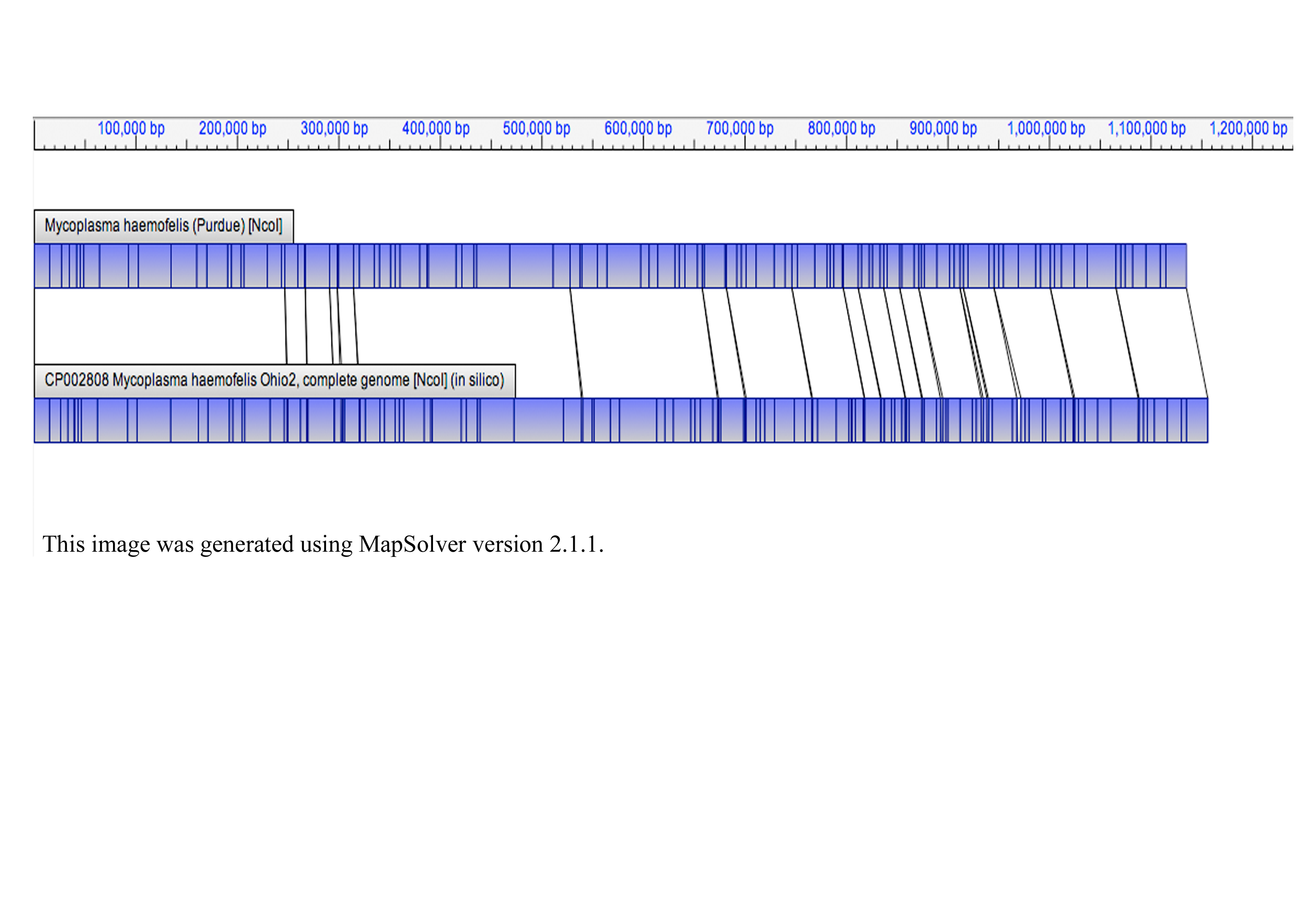

Supplement: Additional file 2 — Figure S2: Validation of the M. haemofelis strain Ohio2 sequence assembly by optical map comparison. Vertical lines represent the restriction site. Regions with similarities are illustrated in blue, regions with differences are illustrated in white. [file 1297-9716-42-102-S2.TIFF]

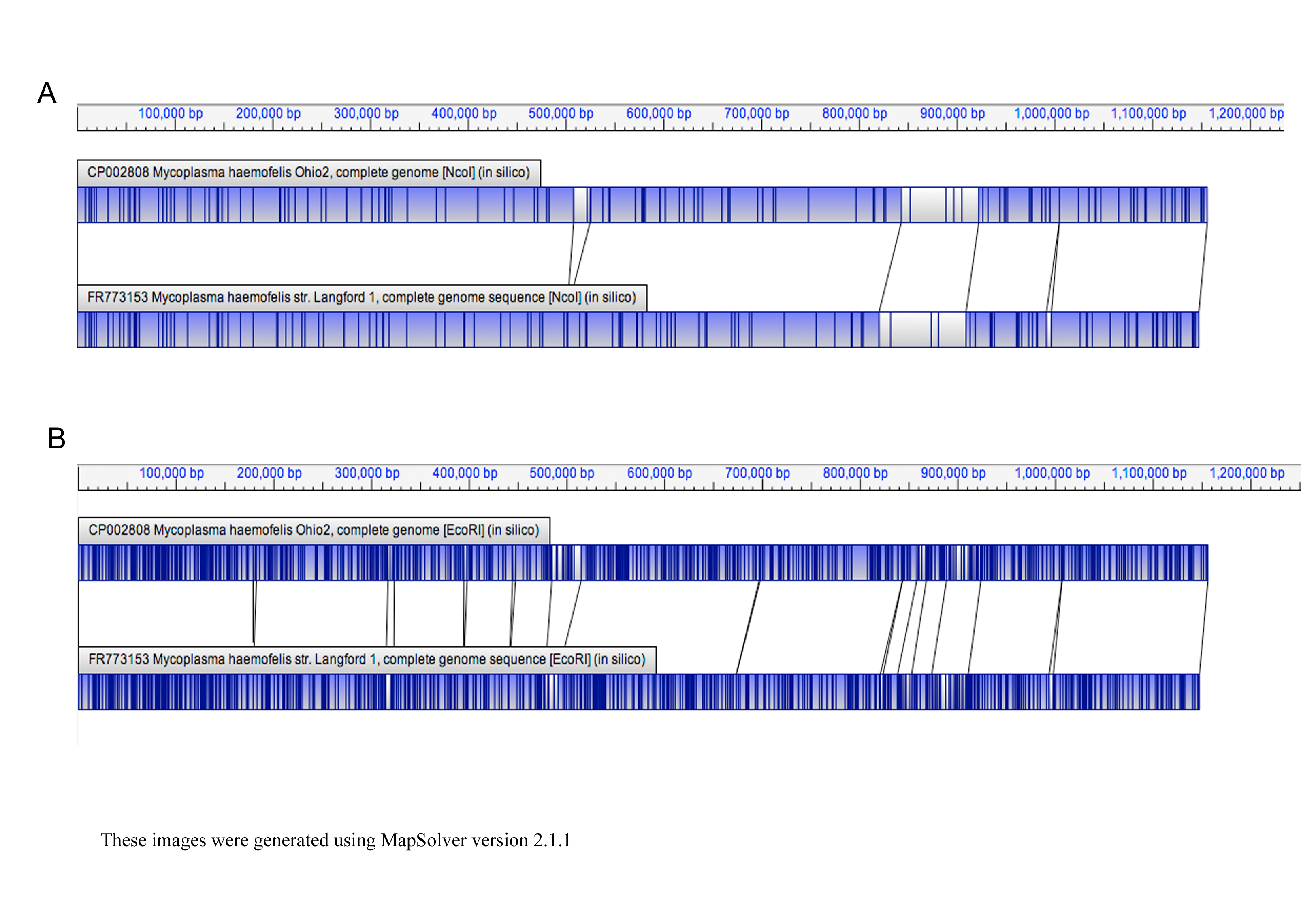

Supplement: Additional file 3 — Figure S3: Comparative analysis of the whole genomes of M. haemofelis strains Ohio2 and Langford 1 by in silico restriction maps. Vertical lines represent the restriction sites. Regions with similarities are illustrated in blue, regions with differences are illustrated in white. (a) Restricted with NcoI (6 cutter), and (b) Restricted with EcoRI (4 cutter). [file 1297-9716-42-102-S3.TIFF]
