# Supplementary material for: Genome of Mycoplasma haemofelis, unraveling its strategies for survival and persistence
Source: Vet Res. 2011 Sep 21;42(1):102. doi: 10.1186/1297-9716-42-102 (PMC3196708; doi:10.1186/1297-9716-42-102)
Supplement: Additional file 7 — Figure S4: Predicted metabolic pathways of M. haemofelis strain Ohio2. Gray boxes represent substrates or products. Gray ellipses are metabolites predicted to be imported from the extracellular environment. White boxes represent enzymes with orthologs in the genome of M. haemofelis. Dashed white boxes represent enzymes with no orthologs in the genome of M. haemofelis. (a) Glycolysis, (b) Nicotinate/Nicotinamide metabolism, (c) Purine metabolism, (d) Pyrimidine metabolism, (e) Lipid metabolism. Pathway predictions were based on KEGG pathway database [35] and the study performed by Yus et al. [36]. [file 1297-9716-42-102-S7.PDF]

(A) Glycolysis

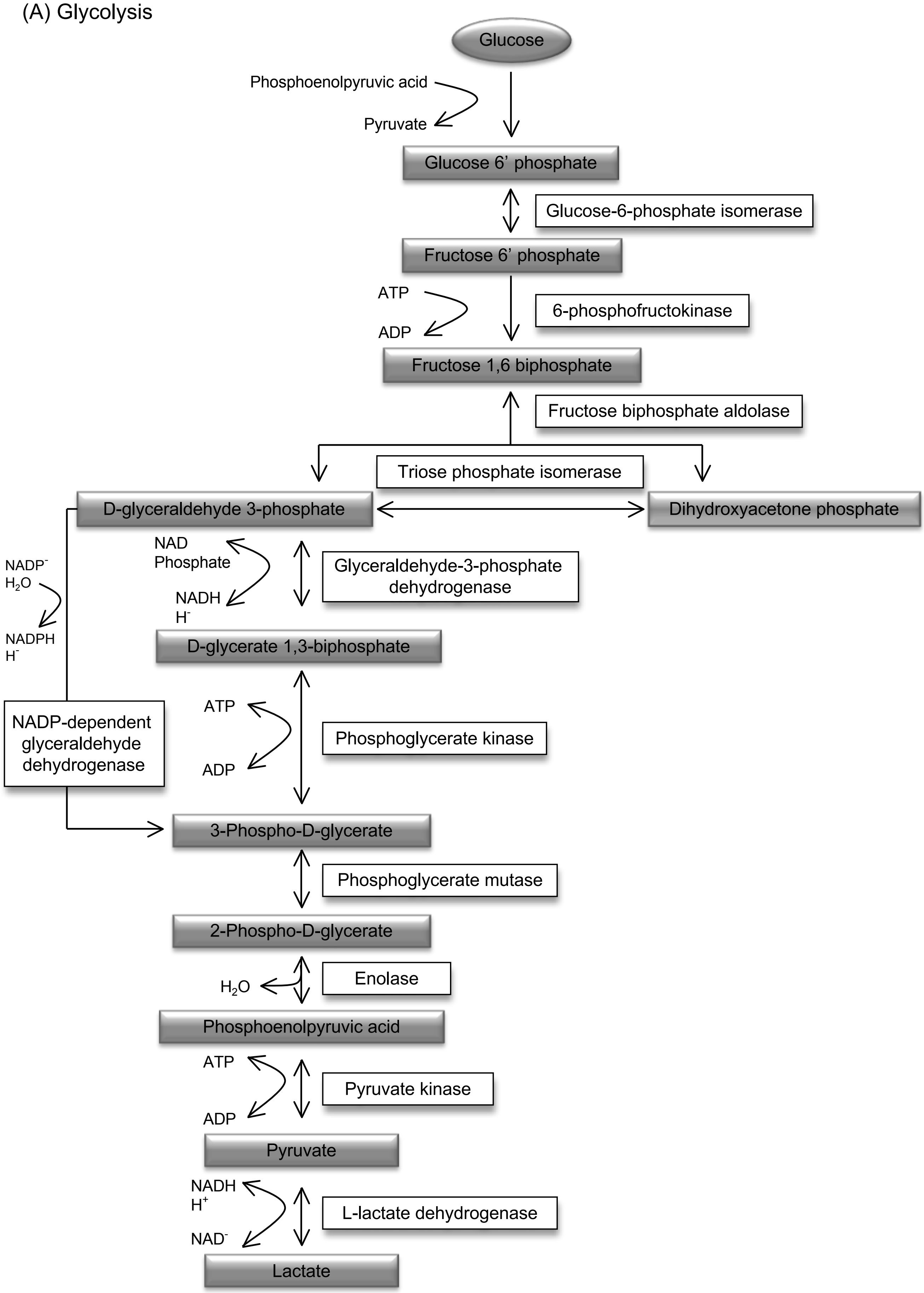

(B) Nicotinate/Nicotinamide Metabolism

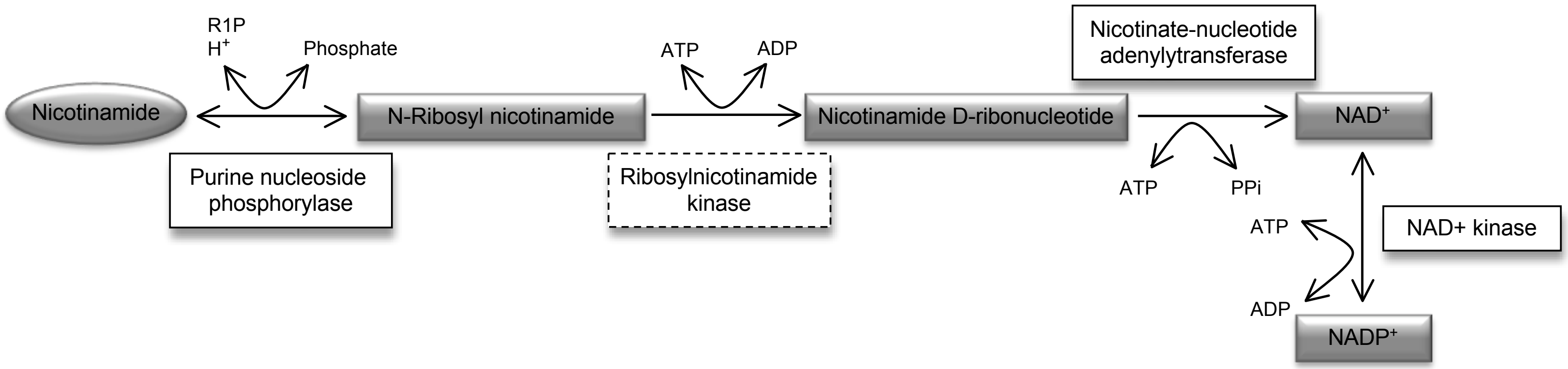

NH_3: Ammonia
PPi: Diphosphate
R1P: Ribose 1' phosphate

(C) Purine Metabolism

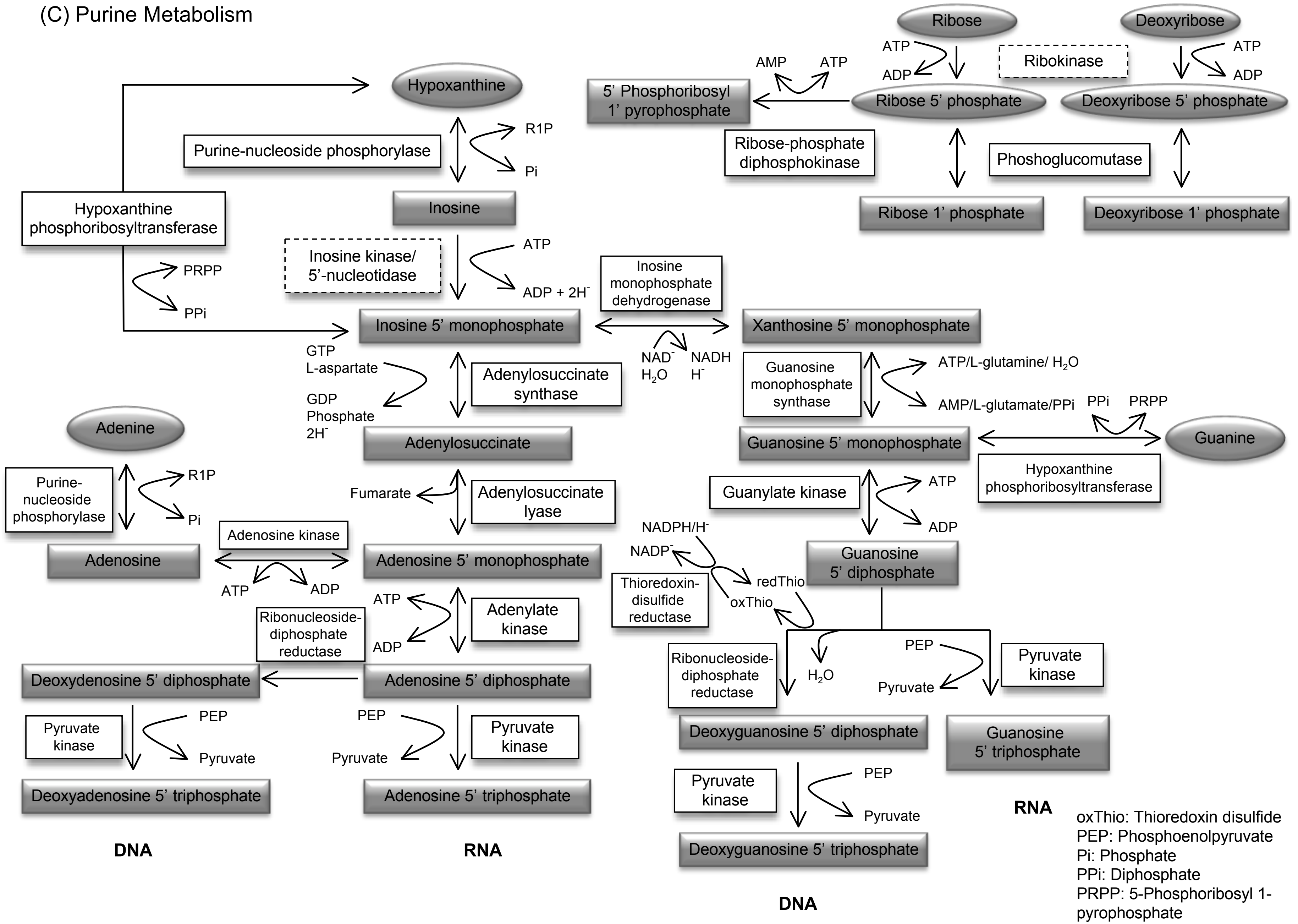

oxThio: Thioredoxin disulfide
PEP: Phosphoenolpyruvate
Pi: Phosphate
PPi: Diphosphate
PRPP: 5-Phosphoribosyl 1-pyrophosphate
R1P: Ribose 1' phosphate
redThio: Thioredoxin

(D) Pyrimidine Metabolism

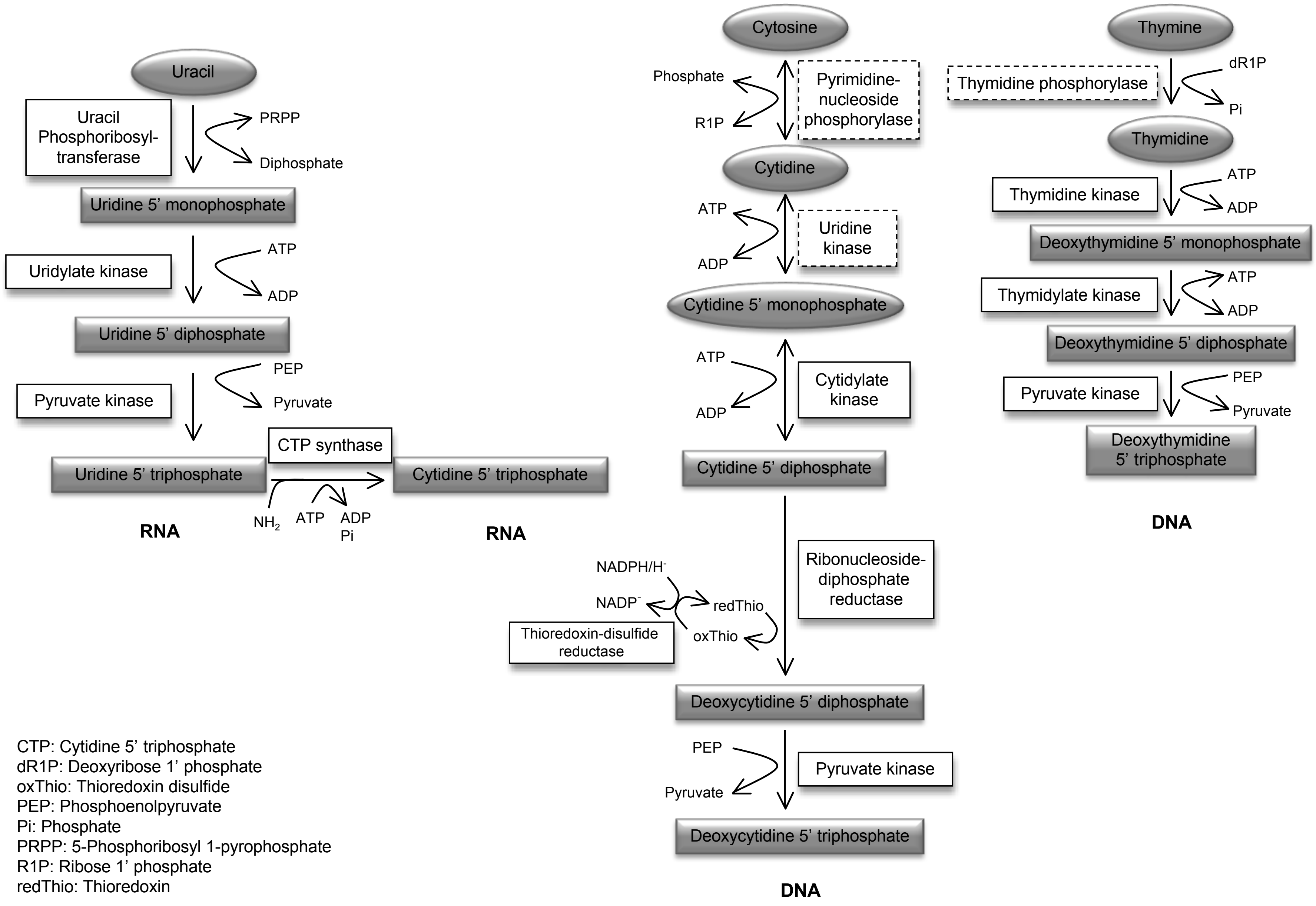

CTP: Cytidine 5’ triphosphate
dR1P: Deoxyribose 1’ phosphate
oxThio: Thioredoxin disulfide
PEP: Phosphoenolpyruvate
Pi: Phosphate
PRPP: 5-Phosphoribosyl 1-pyrophosphate
R1P: Ribose 1’ phosphate
redThio: Thioredoxin

(E) Lipid Metabolism

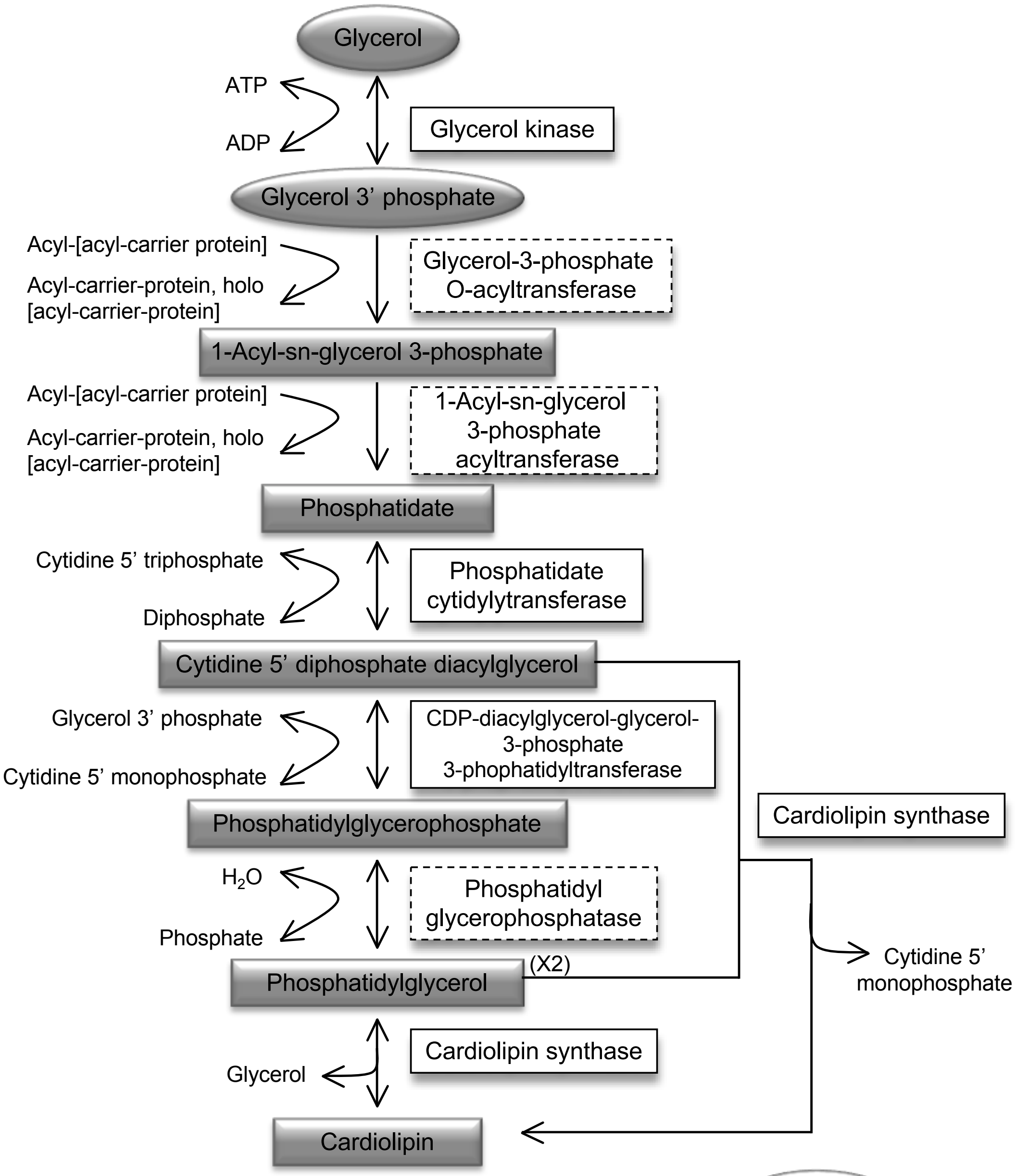

Phospholipid

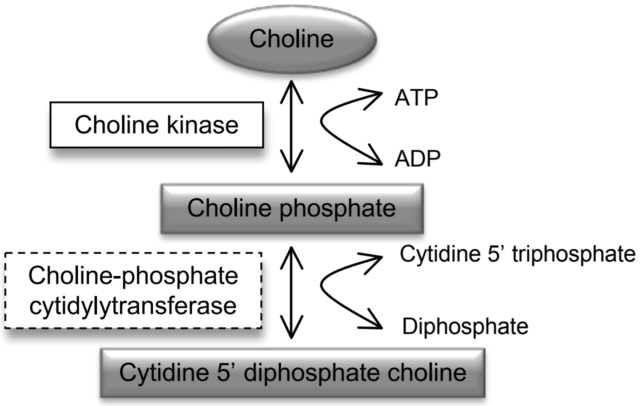

Phospholipid
