# Supplementary material for: Genome of Mycoplasma haemofelis, unraveling its strategies for survival and persistence
Source: Vet Res. 2011 Sep 21;42(1):102. doi: 10.1186/1297-9716-42-102 (PMC3196708; doi:10.1186/1297-9716-42-102)
Supplement: Additional file 9 — Table S5: List of GenBank accession numbers of CDSs of M. haemofelis strain Ohio2 genome distributed in paralog families. This list was generated using the BLASTclust software. [file 1297-9716-42-102-S9.PDF]

Table S5: List of GenBank accession numbers of CDSs of *M. haemofelis* strain Ohio2 genome distributed in paralog families.

Family 1

gi|334193103|gb|AEG72831.1|
gi|334193546|gb|AEG73274.1|
gi|334193805|gb|AEG73533.1|
gi|334193568|gb|AEG73296.1|
gi|334193099|gb|AEG72827.1|
gi|334193549|gb|AEG73277.1|
gi|334193594|gb|AEG73322.1|
gi|334193540|gb|AEG73268.1|
gi|334193556|gb|AEG73284.1|
gi|334193550|gb|AEG73278.1|
gi|334193555|gb|AEG73283.1|
gi|334193560|gb|AEG73288.1|
gi|334193544|gb|AEG73272.1|
gi|334193552|gb|AEG73280.1|
gi|334193553|gb|AEG73281.1|
gi|334193377|gb|AEG73105.1|
gi|334193561|gb|AEG73289.1|
gi|334193357|gb|AEG73085.1|
gi|334193382|gb|AEG73110.1|
gi|334193354|gb|AEG73082.1|
gi|334193281|gb|AEG73009.1|
gi|334193353|gb|AEG73081.1|
gi|334193545|gb|AEG73273.1|
gi|334193258|gb|AEG72986.1|
gi|334193349|gb|AEG73077.1|
gi|334193718|gb|AEG73446.1|
gi|334193373|gb|AEG73101.1|
gi|334193785|gb|AEG73513.1|
gi|334193792|gb|AEG73520.1|
gi|334192870|gb|AEG72598.1|
gi|334192903|gb|AEG72631.1|
gi|334193456|gb|AEG73184.1|
gi|334193845|gb|AEG73573.1|
gi|334193722|gb|AEG73450.1|
gi|334193385|gb|AEG73113.1|
gi|334193880|gb|AEG73608.1|
gi|334192869|gb|AEG72597.1|
gi|334192905|gb|AEG72633.1|
gi|334193488|gb|AEG73216.1|
gi|334192836|gb|AEG72564.1|
gi|334192883|gb|AEG72611.1|

gi|334193466|gb|AEG73194.1|
gi|334192853|gb|AEG72581.1|
gi|334192857|gb|AEG72585.1|
gi|334192874|gb|AEG72602.1|
gi|334192885|gb|AEG72613.1|
gi|334192894|gb|AEG72622.1|
gi|334192895|gb|AEG72623.1|
gi|334193012|gb|AEG72740.1|
gi|334193085|gb|AEG72813.1|
gi|334193496|gb|AEG73224.1|
gi|334193532|gb|AEG73260.1|
gi|334192838|gb|AEG72566.1|
gi|334192882|gb|AEG72610.1|
gi|334192898|gb|AEG72626.1|
gi|334192899|gb|AEG72627.1|
gi|334192900|gb|AEG72628.1|
gi|334192931|gb|AEG72659.1|
gi|334192983|gb|AEG72711.1|
gi|334193502|gb|AEG73230.1|
gi|334193719|gb|AEG73447.1|
gi|334193738|gb|AEG73466.1|
gi|334193742|gb|AEG73470.1|
gi|334193823|gb|AEG73551.1|
gi|334192856|gb|AEG72584.1|
gi|334192873|gb|AEG72601.1|
gi|334192877|gb|AEG72605.1|
gi|334193397|gb|AEG73125.1|
gi|334193713|gb|AEG73441.1|
gi|334193717|gb|AEG73445.1|
gi|334193761|gb|AEG73489.1|
gi|334192901|gb|AEG72629.1|
gi|334192915|gb|AEG72643.1|
gi|334192939|gb|AEG72667.1|
gi|334192943|gb|AEG72671.1|
gi|334192954|gb|AEG72682.1|
gi|334192979|gb|AEG72707.1|
gi|334193711|gb|AEG73439.1|
gi|334193712|gb|AEG73440.1|
gi|334193756|gb|AEG73484.1|
gi|334193927|gb|AEG73655.1|
gi|334192841|gb|AEG72569.1|
gi|334192842|gb|AEG72570.1|
gi|334192845|gb|AEG72573.1|
gi|334192849|gb|AEG72577.1|
gi|334192871|gb|AEG72599.1|
gi|334192875|gb|AEG72603.1|

gi|334192876|gb|AEG72604.1|
gi|334192878|gb|AEG72606.1|
gi|334192881|gb|AEG72609.1|
gi|334192987|gb|AEG72715.1|
gi|334193716|gb|AEG73444.1|
gi|334193849|gb|AEG73577.1|
gi|334193854|gb|AEG73582.1|
gi|334193876|gb|AEG73604.1|
gi|334192837|gb|AEG72565.1|
gi|334192844|gb|AEG72572.1|
gi|334192854|gb|AEG72582.1|
gi|334192868|gb|AEG72596.1|
gi|334192936|gb|AEG72664.1|
gi|334193109|gb|AEG72837.1|
gi|334193497|gb|AEG73225.1|
gi|334193796|gb|AEG73524.1|
gi|334193889|gb|AEG73617.1|
gi|334192848|gb|AEG72576.1|
gi|334192896|gb|AEG72624.1|
gi|334192972|gb|AEG72700.1|
gi|334193352|gb|AEG73080.1|
gi|334193493|gb|AEG73221.1|
gi|334193720|gb|AEG73448.1|
gi|334193757|gb|AEG73485.1|
gi|334193918|gb|AEG73646.1|
gi|334193930|gb|AEG73658.1|
gi|334192858|gb|AEG72586.1|
gi|334192940|gb|AEG72668.1|
gi|334192981|gb|AEG72709.1|
gi|334193440|gb|AEG73168.1|
gi|334193450|gb|AEG73178.1|
gi|334193538|gb|AEG73266.1|
gi|334193745|gb|AEG73473.1|
gi|334193778|gb|AEG73506.1|
gi|334193794|gb|AEG73522.1|
gi|334193885|gb|AEG73613.1|
gi|334193902|gb|AEG73630.1|
gi|334193916|gb|AEG73644.1|
gi|334192855|gb|AEG72583.1|
gi|334193040|gb|AEG72768.1|
gi|334193048|gb|AEG72776.1|
gi|334193374|gb|AEG73102.1|
gi|334193490|gb|AEG73218.1|
gi|334193960|gb|AEG73688.1|
gi|334192760|gb|AEG72488.1|
gi|334192959|gb|AEG72687.1|

gi|334193238|gb|AEG72966.1|
gi|334193408|gb|AEG73136.1|
gi|334193445|gb|AEG73173.1|
gi|334193721|gb|AEG73449.1|
gi|334193958|gb|AEG73686.1|
gi|334193988|gb|AEG73716.1|
gi|334192840|gb|AEG72568.1|
gi|334192884|gb|AEG72612.1|
gi|334192932|gb|AEG72660.1|
gi|334193155|gb|AEG72883.1|
gi|334193350|gb|AEG73078.1|
gi|334193376|gb|AEG73104.1|
gi|334193498|gb|AEG73226.1|
gi|334193714|gb|AEG73442.1|
gi|334193743|gb|AEG73471.1|
gi|334193762|gb|AEG73490.1|
gi|334193824|gb|AEG73552.1|
gi|334193832|gb|AEG73560.1|
gi|334193913|gb|AEG73641.1|
gi|334192735|gb|AEG72463.1|
gi|334192792|gb|AEG72520.1|
gi|334192859|gb|AEG72587.1|
gi|334192917|gb|AEG72645.1|
gi|334192958|gb|AEG72686.1|
gi|334192978|gb|AEG72706.1|
gi|334193210|gb|AEG72938.1|
gi|334193348|gb|AEG73076.1|
gi|334193836|gb|AEG73564.1|
gi|334193838|gb|AEG73566.1|
gi|334193874|gb|AEG73602.1|
gi|334193928|gb|AEG73656.1|
gi|334192740|gb|AEG72468.1|
gi|334192751|gb|AEG72479.1|
gi|334192756|gb|AEG72484.1|
gi|334192764|gb|AEG72492.1|
gi|334192784|gb|AEG72512.1|
gi|334192880|gb|AEG72608.1|
gi|334192935|gb|AEG72663.1|
gi|334193020|gb|AEG72748.1|
gi|334193027|gb|AEG72755.1|
gi|334193253|gb|AEG72981.1|
gi|334193585|gb|AEG73313.1|
gi|334193772|gb|AEG73500.1|
gi|334193818|gb|AEG73546.1|
gi|334193829|gb|AEG73557.1|
gi|334192732|gb|AEG72460.1|

gi|334192763|gb|AEG72491.1|
gi|334192843|gb|AEG72571.1|
gi|334192890|gb|AEG72618.1|
gi|334192908|gb|AEG72636.1|
gi|334192955|gb|AEG72683.1|
gi|334192968|gb|AEG72696.1|
gi|334192980|gb|AEG72708.1|
gi|334192988|gb|AEG72716.1|
gi|334192993|gb|AEG72721.1|
gi|334193001|gb|AEG72729.1|
gi|334193049|gb|AEG72777.1|
gi|334193050|gb|AEG72778.1|
gi|334193131|gb|AEG72859.1|
gi|334193235|gb|AEG72963.1|
gi|334193623|gb|AEG73351.1|
gi|334193867|gb|AEG73595.1|
gi|334193910|gb|AEG73638.1|
gi|334193929|gb|AEG73657.1|
gi|334193939|gb|AEG73667.1|
gi|334193991|gb|AEG73719.1|
gi|334193993|gb|AEG73721.1|
gi|334192766|gb|AEG72494.1|
gi|334192778|gb|AEG72506.1|
gi|334192793|gb|AEG72521.1|
gi|334192803|gb|AEG72531.1|
gi|334192851|gb|AEG72579.1|
gi|334193013|gb|AEG72741.1|
gi|334193019|gb|AEG72747.1|
gi|334193024|gb|AEG72752.1|
gi|334193237|gb|AEG72965.1|
gi|334193261|gb|AEG72989.1|
gi|334193266|gb|AEG72994.1|
gi|334193268|gb|AEG72996.1|
gi|334193478|gb|AEG73206.1|
gi|334193528|gb|AEG73256.1|
gi|334193583|gb|AEG73311.1|
gi|334193764|gb|AEG73492.1|
gi|334193767|gb|AEG73495.1|
gi|334193768|gb|AEG73496.1|
gi|334193771|gb|AEG73499.1|
gi|334193786|gb|AEG73514.1|
gi|334193787|gb|AEG73515.1|
gi|334193841|gb|AEG73569.1|
gi|334193870|gb|AEG73598.1|
gi|334193872|gb|AEG73600.1|
gi|334193898|gb|AEG73626.1|

gi|334193984|gb|AEG73712.1|
gi|334192796|gb|AEG72524.1|
gi|334192800|gb|AEG72528.1|
gi|334192906|gb|AEG72634.1|
gi|334192986|gb|AEG72714.1|
gi|334192996|gb|AEG72724.1|
gi|334193002|gb|AEG72730.1|
gi|334193041|gb|AEG72769.1|
gi|334193045|gb|AEG72773.1|
gi|334193057|gb|AEG72785.1|
gi|334193127|gb|AEG72855.1|
gi|334193409|gb|AEG73137.1|
gi|334193419|gb|AEG73147.1|
gi|334193429|gb|AEG73157.1|
gi|334193447|gb|AEG73175.1|
gi|334193458|gb|AEG73186.1|
gi|334193580|gb|AEG73308.1|
gi|334193604|gb|AEG73332.1|
gi|334193735|gb|AEG73463.1|
gi|334193760|gb|AEG73488.1|
gi|334193847|gb|AEG73575.1|
gi|334193852|gb|AEG73580.1|
gi|334193869|gb|AEG73597.1|
gi|334193873|gb|AEG73601.1|
gi|334193875|gb|AEG73603.1|
gi|334193919|gb|AEG73647.1|
gi|334193963|gb|AEG73691.1|
gi|334193964|gb|AEG73692.1|
gi|334192755|gb|AEG72483.1|
gi|334192779|gb|AEG72507.1|
gi|334192794|gb|AEG72522.1|
gi|334192925|gb|AEG72653.1|
gi|334192969|gb|AEG72697.1|
gi|334193000|gb|AEG72728.1|
gi|334193016|gb|AEG72744.1|
gi|334193031|gb|AEG72759.1|
gi|334193047|gb|AEG72775.1|
gi|334193055|gb|AEG72783.1|
gi|334193074|gb|AEG72802.1|
gi|334193078|gb|AEG72806.1|
gi|334193087|gb|AEG72815.1|
gi|334193152|gb|AEG72880.1|
gi|334193153|gb|AEG72881.1|
gi|334193186|gb|AEG72914.1|
gi|334193187|gb|AEG72915.1|
gi|334193251|gb|AEG72979.1|

gi|334193400|gb|AEG73128.1|
gi|334193431|gb|AEG73159.1|
gi|334193480|gb|AEG73208.1|
gi|334193504|gb|AEG73232.1|
gi|334193514|gb|AEG73242.1|
gi|334193739|gb|AEG73467.1|
gi|334193753|gb|AEG73481.1|
gi|334193882|gb|AEG73610.1|
gi|334193909|gb|AEG73637.1|
gi|334193912|gb|AEG73640.1|
gi|334193915|gb|AEG73643.1|
gi|334193966|gb|AEG73694.1|
gi|334192752|gb|AEG72480.1|
gi|334192759|gb|AEG72487.1|
gi|334192783|gb|AEG72511.1|
gi|334192846|gb|AEG72574.1|
gi|334192852|gb|AEG72580.1|
gi|334192907|gb|AEG72635.1|
gi|334192909|gb|AEG72637.1|
gi|334192994|gb|AEG72722.1|
gi|334193009|gb|AEG72737.1|
gi|334193063|gb|AEG72791.1|
gi|334193070|gb|AEG72798.1|
gi|334193075|gb|AEG72803.1|
gi|334193119|gb|AEG72847.1|
gi|334193233|gb|AEG72961.1|
gi|334193392|gb|AEG73120.1|
gi|334193396|gb|AEG73124.1|
gi|334193414|gb|AEG73142.1|
gi|334193452|gb|AEG73180.1|
gi|334193475|gb|AEG73203.1|
gi|334193621|gb|AEG73349.1|
gi|334193813|gb|AEG73541.1|
gi|334193924|gb|AEG73652.1|
gi|334193932|gb|AEG73660.1|
gi|334193933|gb|AEG73661.1|
gi|334193944|gb|AEG73672.1|
gi|334193962|gb|AEG73690.1|
gi|334193971|gb|AEG73699.1|
gi|334193979|gb|AEG73707.1|
gi|334193980|gb|AEG73708.1|
gi|334192754|gb|AEG72482.1|
gi|334192777|gb|AEG72505.1|
gi|334192807|gb|AEG72535.1|
gi|334192861|gb|AEG72589.1|
gi|334192902|gb|AEG72630.1|

gi|334192927|gb|AEG72655.1|
gi|334192975|gb|AEG72703.1|
gi|334193026|gb|AEG72754.1|
gi|334193051|gb|AEG72779.1|
gi|334193071|gb|AEG72799.1|
gi|334193160|gb|AEG72888.1|
gi|334193163|gb|AEG72891.1|
gi|334193173|gb|AEG72901.1|
gi|334193345|gb|AEG73073.1|
gi|334193421|gb|AEG73149.1|
gi|334193748|gb|AEG73476.1|
gi|334193751|gb|AEG73479.1|
gi|334193758|gb|AEG73486.1|
gi|334193793|gb|AEG73521.1|
gi|334193901|gb|AEG73629.1|
gi|334193926|gb|AEG73654.1|
gi|334193934|gb|AEG73662.1|
gi|334193967|gb|AEG73695.1|
gi|334192765|gb|AEG72493.1|
gi|334192795|gb|AEG72523.1|
gi|334192850|gb|AEG72578.1|
gi|334192862|gb|AEG72590.1|
gi|334192892|gb|AEG72620.1|
gi|334193005|gb|AEG72733.1|
gi|334193006|gb|AEG72734.1|
gi|334193010|gb|AEG72738.1|
gi|334193054|gb|AEG72782.1|
gi|334193084|gb|AEG72812.1|
gi|334193111|gb|AEG72839.1|
gi|334193249|gb|AEG72977.1|
gi|334193406|gb|AEG73134.1|
gi|334193451|gb|AEG73179.1|
gi|334193463|gb|AEG73191.1|
gi|334193588|gb|AEG73316.1|
gi|334193616|gb|AEG73344.1|
gi|334193737|gb|AEG73465.1|
gi|334193747|gb|AEG73475.1|
gi|334193769|gb|AEG73497.1|
gi|334193777|gb|AEG73505.1|
gi|334193816|gb|AEG73544.1|
gi|334193833|gb|AEG73561.1|
gi|334193835|gb|AEG73563.1|
gi|334193851|gb|AEG73579.1|
gi|334193968|gb|AEG73696.1|
gi|334192891|gb|AEG72619.1|
gi|334192956|gb|AEG72684.1|

gi|334192999|gb|AEG72727.1|
gi|334193007|gb|AEG72735.1|
gi|334193008|gb|AEG72736.1|
gi|334193023|gb|AEG72751.1|
gi|334193044|gb|AEG72772.1|
gi|334193058|gb|AEG72786.1|
gi|334193091|gb|AEG72819.1|
gi|334193118|gb|AEG72846.1|
gi|334193157|gb|AEG72885.1|
gi|334193182|gb|AEG72910.1|
gi|334193250|gb|AEG72978.1|
gi|334193371|gb|AEG73099.1|
gi|334193428|gb|AEG73156.1|
gi|334193430|gb|AEG73158.1|
gi|334193432|gb|AEG73160.1|
gi|334193434|gb|AEG73162.1|
gi|334193479|gb|AEG73207.1|
gi|334193515|gb|AEG73243.1|
gi|334193774|gb|AEG73502.1|
gi|334193856|gb|AEG73584.1|
gi|334193877|gb|AEG73605.1|
gi|334193884|gb|AEG73612.1|
gi|334193922|gb|AEG73650.1|
gi|334193973|gb|AEG73701.1|
gi|334193982|gb|AEG73710.1|
gi|334192743|gb|AEG72471.1|
gi|334192804|gb|AEG72532.1|
gi|334192974|gb|AEG72702.1|
gi|334193032|gb|AEG72760.1|
gi|334193062|gb|AEG72790.1|
gi|334193080|gb|AEG72808.1|
gi|334193094|gb|AEG72822.1|
gi|334193106|gb|AEG72834.1|
gi|334193159|gb|AEG72887.1|
gi|334193395|gb|AEG73123.1|
gi|334193426|gb|AEG73154.1|
gi|334193501|gb|AEG73229.1|
gi|334193512|gb|AEG73240.1|
gi|334193521|gb|AEG73249.1|
gi|334193523|gb|AEG73251.1|
gi|334193749|gb|AEG73477.1|
gi|334193750|gb|AEG73478.1|
gi|334193888|gb|AEG73616.1|
gi|334193900|gb|AEG73628.1|
gi|334193904|gb|AEG73632.1|
gi|334193920|gb|AEG73648.1|

gi|334193954|gb|AEG73682.1|
gi|334193986|gb|AEG73714.1|
gi|334192741|gb|AEG72469.1|
gi|334192748|gb|AEG72476.1|
gi|334192786|gb|AEG72514.1|
gi|334192791|gb|AEG72519.1|
gi|334192864|gb|AEG72592.1|
gi|334192879|gb|AEG72607.1|
gi|334192914|gb|AEG72642.1|
gi|334192933|gb|AEG72661.1|
gi|334192964|gb|AEG72692.1|
gi|334192995|gb|AEG72723.1|
gi|334192998|gb|AEG72726.1|
gi|334193025|gb|AEG72753.1|
gi|334193056|gb|AEG72784.1|
gi|334193066|gb|AEG72794.1|
gi|334193082|gb|AEG72810.1|
gi|334193144|gb|AEG72872.1|
gi|334193256|gb|AEG72984.1|
gi|334193407|gb|AEG73135.1|
gi|334193411|gb|AEG73139.1|
gi|334193420|gb|AEG73148.1|
gi|334193442|gb|AEG73170.1|
gi|334193465|gb|AEG73193.1|
gi|334193468|gb|AEG73196.1|
gi|334193473|gb|AEG73201.1|
gi|334193489|gb|AEG73217.1|
gi|334193505|gb|AEG73233.1|
gi|334193518|gb|AEG73246.1|
gi|334193534|gb|AEG73262.1|
gi|334193539|gb|AEG73267.1|
gi|334193578|gb|AEG73306.1|
gi|334193600|gb|AEG73328.1|
gi|334193755|gb|AEG73483.1|
gi|334193766|gb|AEG73494.1|
gi|334193797|gb|AEG73525.1|
gi|334193840|gb|AEG73568.1|
gi|334193860|gb|AEG73588.1|
gi|334193878|gb|AEG73606.1|
gi|334193883|gb|AEG73611.1|
gi|334193905|gb|AEG73633.1|
gi|334193917|gb|AEG73645.1|
gi|334193921|gb|AEG73649.1|
gi|334193946|gb|AEG73674.1|
gi|334193955|gb|AEG73683.1|
gi|334193972|gb|AEG73700.1|

gi|334192787|gb|AEG72515.1|
gi|334192912|gb|AEG72640.1|
gi|334192944|gb|AEG72672.1|
gi|334192990|gb|AEG72718.1|
gi|334193029|gb|AEG72757.1|
gi|334193079|gb|AEG72807.1|
gi|334193093|gb|AEG72821.1|
gi|334193105|gb|AEG72833.1|
gi|334193132|gb|AEG72860.1|
gi|334193133|gb|AEG72861.1|
gi|334193154|gb|AEG72882.1|
gi|334193212|gb|AEG72940.1|
gi|334193254|gb|AEG72982.1|
gi|334193260|gb|AEG72988.1|
gi|334193370|gb|AEG73098.1|
gi|334193379|gb|AEG73107.1|
gi|334193393|gb|AEG73121.1|
gi|334193472|gb|AEG73200.1|
gi|334193483|gb|AEG73211.1|
gi|334193510|gb|AEG73238.1|
gi|334193524|gb|AEG73252.1|
gi|334193576|gb|AEG73304.1|
gi|334193577|gb|AEG73305.1|
gi|334193618|gb|AEG73346.1|
gi|334193741|gb|AEG73469.1|
gi|334193828|gb|AEG73556.1|
gi|334193865|gb|AEG73593.1|
gi|334193871|gb|AEG73599.1|
gi|334193907|gb|AEG73635.1|
gi|334193914|gb|AEG73642.1|
gi|334193923|gb|AEG73651.1|
gi|334193940|gb|AEG73668.1|
gi|334193948|gb|AEG73676.1|
gi|334193950|gb|AEG73678.1|
gi|334193956|gb|AEG73684.1|
gi|334193989|gb|AEG73717.1|
gi|334192913|gb|AEG72641.1|
gi|334192950|gb|AEG72678.1|
gi|334192962|gb|AEG72690.1|
gi|334192992|gb|AEG72720.1|
gi|334193015|gb|AEG72743.1|
gi|334193035|gb|AEG72763.1|
gi|334193067|gb|AEG72795.1|
gi|334193121|gb|AEG72849.1|
gi|334193129|gb|AEG72857.1|
gi|334193136|gb|AEG72864.1|

gi|334193151|gb|AEG72879.1|
gi|334193196|gb|AEG72924.1|
gi|334193252|gb|AEG72980.1|
gi|334193383|gb|AEG73111.1|
gi|334193384|gb|AEG73112.1|
gi|334193433|gb|AEG73161.1|
gi|334193436|gb|AEG73164.1|
gi|334193441|gb|AEG73169.1|
gi|334193487|gb|AEG73215.1|
gi|334193516|gb|AEG73244.1|
gi|334193519|gb|AEG73247.1|
gi|334193530|gb|AEG73258.1|
gi|334193533|gb|AEG73261.1|
gi|334193551|gb|AEG73279.1|
gi|334193586|gb|AEG73314.1|
gi|334193744|gb|AEG73472.1|
gi|334193776|gb|AEG73504.1|
gi|334193820|gb|AEG73548.1|
gi|334193821|gb|AEG73549.1|
gi|334193822|gb|AEG73550.1|
gi|334193846|gb|AEG73574.1|
gi|334193879|gb|AEG73607.1|
gi|334193887|gb|AEG73615.1|
gi|334193897|gb|AEG73625.1|
gi|334193969|gb|AEG73697.1|
gi|334193974|gb|AEG73702.1|
gi|334193978|gb|AEG73706.1|
gi|334193990|gb|AEG73718.1|
gi|334192790|gb|AEG72518.1|
gi|334192923|gb|AEG72651.1|
gi|334192942|gb|AEG72670.1|
gi|334192960|gb|AEG72688.1|
gi|334192977|gb|AEG72705.1|
gi|334193042|gb|AEG72770.1|
gi|334193052|gb|AEG72780.1|
gi|334193143|gb|AEG72871.1|
gi|334193164|gb|AEG72892.1|
gi|334193181|gb|AEG72909.1|
gi|334193240|gb|AEG72968.1|
gi|334193359|gb|AEG73087.1|
gi|334193375|gb|AEG73103.1|
gi|334193402|gb|AEG73130.1|
gi|334193423|gb|AEG73151.1|
gi|334193457|gb|AEG73185.1|
gi|334193459|gb|AEG73187.1|
gi|334193461|gb|AEG73189.1|

gi|334193471|gb|AEG73199.1|
gi|334193507|gb|AEG73235.1|
gi|334193511|gb|AEG73239.1|
gi|334193513|gb|AEG73241.1|
gi|334193602|gb|AEG73330.1|
gi|334193619|gb|AEG73347.1|
gi|334193819|gb|AEG73547.1|
gi|334193953|gb|AEG73681.1|
gi|334193977|gb|AEG73705.1|
gi|334192926|gb|AEG72654.1|
gi|334192938|gb|AEG72666.1|
gi|334192945|gb|AEG72673.1|
gi|334193030|gb|AEG72758.1|
gi|334193060|gb|AEG72788.1|
gi|334193065|gb|AEG72793.1|
gi|334193122|gb|AEG72850.1|
gi|334193189|gb|AEG72917.1|
gi|334193220|gb|AEG72948.1|
gi|334193245|gb|AEG72973.1|
gi|334193346|gb|AEG73074.1|
gi|334193347|gb|AEG73075.1|
gi|334193358|gb|AEG73086.1|
gi|334193390|gb|AEG73118.1|
gi|334193455|gb|AEG73183.1|
gi|334193476|gb|AEG73204.1|
gi|334193482|gb|AEG73210.1|
gi|334193486|gb|AEG73214.1|
gi|334193503|gb|AEG73231.1|
gi|334193506|gb|AEG73234.1|
gi|334193509|gb|AEG73237.1|
gi|334193520|gb|AEG73248.1|
gi|334193526|gb|AEG73254.1|
gi|334193527|gb|AEG73255.1|
gi|334193536|gb|AEG73264.1|
gi|334193579|gb|AEG73307.1|
gi|334193613|gb|AEG73341.1|
gi|334193825|gb|AEG73553.1|
gi|334193886|gb|AEG73614.1|
gi|334193975|gb|AEG73703.1|
gi|334192788|gb|AEG72516.1|
gi|334192930|gb|AEG72658.1|
gi|334192985|gb|AEG72713.1|
gi|334193064|gb|AEG72792.1|
gi|334193120|gb|AEG72848.1|
gi|334193135|gb|AEG72863.1|
gi|334193367|gb|AEG73095.1|

gi|334193372|gb|AEG73100.1|
gi|334193380|gb|AEG73108.1|
gi|334193424|gb|AEG73152.1|
gi|334193467|gb|AEG73195.1|
gi|334193477|gb|AEG73205.1|
gi|334193481|gb|AEG73209.1|
gi|334193484|gb|AEG73212.1|
gi|334193541|gb|AEG73269.1|
gi|334193557|gb|AEG73285.1|
gi|334193601|gb|AEG73329.1|
gi|334193617|gb|AEG73345.1|
gi|334193763|gb|AEG73491.1|
gi|334193770|gb|AEG73498.1|
gi|334193896|gb|AEG73624.1|
gi|334193943|gb|AEG73671.1|
gi|334193947|gb|AEG73675.1|
gi|334193951|gb|AEG73679.1|
gi|334192749|gb|AEG72477.1|
gi|334192916|gb|AEG72644.1|
gi|334192922|gb|AEG72650.1|
gi|334193003|gb|AEG72731.1|
gi|334193014|gb|AEG72742.1|
gi|334193138|gb|AEG72866.1|
gi|334193183|gb|AEG72911.1|
gi|334193205|gb|AEG72933.1|
gi|334193234|gb|AEG72962.1|
gi|334193265|gb|AEG72993.1|
gi|334193351|gb|AEG73079.1|
gi|334193356|gb|AEG73084.1|
gi|334193368|gb|AEG73096.1|
gi|334193391|gb|AEG73119.1|
gi|334193413|gb|AEG73141.1|
gi|334193422|gb|AEG73150.1|
gi|334193474|gb|AEG73202.1|
gi|334193517|gb|AEG73245.1|
gi|334193522|gb|AEG73250.1|
gi|334193554|gb|AEG73282.1|
gi|334193581|gb|AEG73309.1|
gi|334193858|gb|AEG73586.1|
gi|334193908|gb|AEG73636.1|
gi|334193945|gb|AEG73673.1|
gi|334193949|gb|AEG73677.1|
gi|334193981|gb|AEG73709.1|
gi|334193987|gb|AEG73715.1|
gi|334192742|gb|AEG72470.1|
gi|334192785|gb|AEG72513.1|

gi|334192946|gb|AEG72674.1|
gi|334192957|gb|AEG72685.1|
gi|334192976|gb|AEG72704.1|
gi|334193076|gb|AEG72804.1|
gi|334193125|gb|AEG72853.1|
gi|334193150|gb|AEG72878.1|
gi|334193165|gb|AEG72893.1|
gi|334193177|gb|AEG72905.1|
gi|334193188|gb|AEG72916.1|
gi|334193191|gb|AEG72919.1|
gi|334193193|gb|AEG72921.1|
gi|334193344|gb|AEG73072.1|
gi|334193378|gb|AEG73106.1|
gi|334193404|gb|AEG73132.1|
gi|334193427|gb|AEG73155.1|
gi|334193605|gb|AEG73333.1|
gi|334193622|gb|AEG73350.1|
gi|334193765|gb|AEG73493.1|
gi|334193834|gb|AEG73562.1|
gi|334193866|gb|AEG73594.1|
gi|334193899|gb|AEG73627.1|
gi|334193911|gb|AEG73639.1|
gi|334193959|gb|AEG73687.1|
gi|334192746|gb|AEG72474.1|
gi|334192953|gb|AEG72681.1|
gi|334193022|gb|AEG72750.1|
gi|334193061|gb|AEG72789.1|
gi|334193069|gb|AEG72797.1|
gi|334193073|gb|AEG72801.1|
gi|334193092|gb|AEG72820.1|
gi|334193343|gb|AEG73071.1|
gi|334193369|gb|AEG73097.1|
gi|334193464|gb|AEG73192.1|
gi|334193485|gb|AEG73213.1|
gi|334193535|gb|AEG73263.1|
gi|334193542|gb|AEG73270.1|
gi|334193620|gb|AEG73348.1|
gi|334193775|gb|AEG73503.1|
gi|334193011|gb|AEG72739.1|
gi|334193072|gb|AEG72800.1|
gi|334193123|gb|AEG72851.1|
gi|334193355|gb|AEG73083.1|
gi|334193381|gb|AEG73109.1|
gi|334193410|gb|AEG73138.1|
gi|334193415|gb|AEG73143.1|
gi|334193425|gb|AEG73153.1|

gi|334193435|gb|AEG73163.1|
gi|334193500|gb|AEG73228.1|
gi|334193525|gb|AEG73253.1|
gi|334193587|gb|AEG73315.1|
gi|334193759|gb|AEG73487.1|
gi|334193843|gb|AEG73571.1|
gi|334193941|gb|AEG73669.1|
gi|334193965|gb|AEG73693.1|
gi|334192941|gb|AEG72669.1|
gi|334193068|gb|AEG72796.1|
gi|334193104|gb|AEG72832.1|
gi|334193140|gb|AEG72868.1|
gi|334193184|gb|AEG72912.1|
gi|334193394|gb|AEG73122.1|
gi|334193603|gb|AEG73331.1|
gi|334193752|gb|AEG73480.1|
gi|334192782|gb|AEG72510.1|
gi|334192924|gb|AEG72652.1|
gi|334193529|gb|AEG73257.1|
gi|334193582|gb|AEG73310.1|
gi|334193615|gb|AEG73343.1|
gi|334193740|gb|AEG73468.1|
gi|334192934|gb|AEG72662.1|
gi|334193156|gb|AEG72884.1|
gi|334193491|gb|AEG73219.1|
gi|334193508|gb|AEG73236.1|
gi|334193783|gb|AEG73511.1|
gi|334193903|gb|AEG73631.1|
gi|334192810|gb|AEG72538.1|
gi|334193124|gb|AEG72852.1|
gi|334193176|gb|AEG72904.1|
gi|334193190|gb|AEG72918.1|
gi|334193194|gb|AEG72922.1|
gi|334193815|gb|AEG73543.1|
gi|334192982|gb|AEG72710.1|
gi|334192997|gb|AEG72725.1|
gi|334193036|gb|AEG72764.1|
gi|334193077|gb|AEG72805.1|
gi|334193081|gb|AEG72809.1|
gi|334193790|gb|AEG73518.1|
gi|334193931|gb|AEG73659.1|
gi|334192758|gb|AEG72486.1|
gi|334193053|gb|AEG72781.1|
gi|334193213|gb|AEG72941.1|
gi|334193531|gb|AEG73259.1|
gi|334192949|gb|AEG72677.1|

gi|334193412|gb|AEG73140.1|
gi|334193881|gb|AEG73609.1|
gi|334193970|gb|AEG73698.1|
gi|334192802|gb|AEG72530.1|
gi|334192809|gb|AEG72537.1|
gi|334192920|gb|AEG72648.1|
gi|334192951|gb|AEG72679.1|
gi|334193004|gb|AEG72732.1|
gi|334193460|gb|AEG73188.1|
gi|334193614|gb|AEG73342.1|
gi|334192745|gb|AEG72473.1|
gi|334192781|gb|AEG72509.1|
gi|334192799|gb|AEG72527.1|
gi|334192806|gb|AEG72534.1|
gi|334193198|gb|AEG72926.1|
gi|334193217|gb|AEG72945.1|
gi|334193236|gb|AEG72964.1|
gi|334192753|gb|AEG72481.1|
gi|334192904|gb|AEG72632.1|
gi|334193166|gb|AEG72894.1|
gi|334193202|gb|AEG72930.1|
gi|334193942|gb|AEG73670.1|
gi|334193134|gb|AEG72862.1|
gi|334193162|gb|AEG72890.1|
gi|334193401|gb|AEG73129.1|
gi|334193976|gb|AEG73704.1|
gi|334192801|gb|AEG72529.1|
gi|334193262|gb|AEG72990.1|
gi|334193126|gb|AEG72854.1|
gi|334192780|gb|AEG72508.1|
gi|334193192|gb|AEG72920.1|
gi|334192744|gb|AEG72472.1|
gi|334192750|gb|AEG72478.1|
gi|334192757|gb|AEG72485.1|
gi|334192805|gb|AEG72533.1|
gi|334192847|gb|AEG72575.1|
gi|334192897|gb|AEG72625.1|
gi|334193021|gb|AEG72749.1|
gi|334193439|gb|AEG73167.1|
gi|334193795|gb|AEG73523.1|
gi|334192970|gb|AEG72698.1|
gi|334193470|gb|AEG73198.1|
gi|334192947|gb|AEG72675.1|
gi|334193492|gb|AEG73220.1|
gi|334193398|gb|AEG73126.1|
gi|334192863|gb|AEG72591.1|

gi|334193754|gb|AEG73482.1|
gi|334193839|gb|AEG73567.1|
gi|334193788|gb|AEG73516.1|
gi|334193837|gb|AEG73565.1|
gi|334193791|gb|AEG73519.1|
gi|334193773|gb|AEG73501.1|
gi|334193784|gb|AEG73512.1|
gi|334193842|gb|AEG73570.1|
gi|334193844|gb|AEG73572.1|
gi|334193848|gb|AEG73576.1|
gi|334193853|gb|AEG73581.1|
gi|334193850|gb|AEG73578.1|
gi|334193855|gb|AEG73583.1|
gi|334193779|gb|AEG73507.1|
gi|334193789|gb|AEG73517.1|
gi|334193444|gb|AEG73172.1|
gi|334193462|gb|AEG73190.1|
gi|334193537|gb|AEG73265.1|
gi|334193043|gb|AEG72771.1|
gi|334192839|gb|AEG72567.1|
gi|334193403|gb|AEG73131.1|
gi|334193857|gb|AEG73585.1|
gi|334193734|gb|AEG73462.1|

Family 2

---

gi|334193231|gb|AEG72959.1|
gi|334193670|gb|AEG73398.1|
gi|334193664|gb|AEG73392.1|
gi|334193673|gb|AEG73401.1|
gi|334193227|gb|AEG72955.1|
gi|334193660|gb|AEG73388.1|
gi|334193679|gb|AEG73407.1|
gi|334193203|gb|AEG72931.1|
gi|334193117|gb|AEG72845.1|
gi|334193675|gb|AEG73403.1|
gi|334193659|gb|AEG73387.1|
gi|334193668|gb|AEG73396.1|
gi|334193230|gb|AEG72958.1|
gi|334193116|gb|AEG72844.1|
gi|334193224|gb|AEG72952.1|
gi|334193631|gb|AEG73359.1|
gi|334193665|gb|AEG73393.1|
gi|334193223|gb|AEG72951.1|
gi|334193229|gb|AEG72957.1|
gi|334193200|gb|AEG72928.1|

gi|334193639|gb|AEG73367.1|
gi|334193228|gb|AEG72956.1|
gi|334193676|gb|AEG73404.1|
gi|334193226|gb|AEG72954.1|
gi|334193669|gb|AEG73397.1|
gi|334193222|gb|AEG72950.1|
gi|334193642|gb|AEG73370.1|
gi|334193225|gb|AEG72953.1|
gi|334193627|gb|AEG73355.1|
gi|334193635|gb|AEG73363.1|
gi|334193624|gb|AEG73352.1|
gi|334193677|gb|AEG73405.1|
gi|334193671|gb|AEG73399.1|
gi|334193672|gb|AEG73400.1|
gi|334193221|gb|AEG72949.1|
gi|334193232|gb|AEG72960.1|
gi|334193264|gb|AEG72992.1|
gi|334193175|gb|AEG72903.1|
gi|334193667|gb|AEG73395.1|
gi|334193171|gb|AEG72899.1|
gi|334193610|gb|AEG73338.1|
gi|334193632|gb|AEG73360.1|
gi|334193646|gb|AEG73374.1|
gi|334193655|gb|AEG73383.1|
gi|334193651|gb|AEG73379.1|
gi|334193634|gb|AEG73362.1|
gi|334193648|gb|AEG73376.1|
gi|334193682|gb|AEG73410.1|
gi|334193199|gb|AEG72927.1|
gi|334193680|gb|AEG73408.1|
gi|334193666|gb|AEG73394.1|
gi|334193683|gb|AEG73411.1|
gi|334193170|gb|AEG72898.1|
gi|334193657|gb|AEG73385.1|
gi|334193658|gb|AEG73386.1|
gi|334193662|gb|AEG73390.1|
gi|334193626|gb|AEG73354.1|
gi|334193629|gb|AEG73357.1|
gi|334193201|gb|AEG72929.1|
gi|334193625|gb|AEG73353.1|
gi|334193098|gb|AEG72826.1|
gi|334193606|gb|AEG73334.1|
gi|334193628|gb|AEG73356.1|
gi|334193644|gb|AEG73372.1|
gi|334193654|gb|AEG73382.1|
gi|334193798|gb|AEG73526.1|

gi|334193114|gb|AEG72842.1|
gi|334193128|gb|AEG72856.1|
gi|334193113|gb|AEG72841.1|
gi|334193574|gb|AEG73302.1|
gi|334193678|gb|AEG73406.1|
gi|334193607|gb|AEG73335.1|
gi|334193661|gb|AEG73389.1|
gi|334193174|gb|AEG72902.1|
gi|334193681|gb|AEG73409.1|
gi|334193267|gb|AEG72995.1|
gi|334193263|gb|AEG72991.1|
gi|334193284|gb|AEG73012.1|
gi|334192967|gb|AEG72695.1|
gi|334193242|gb|AEG72970.1|
gi|334193248|gb|AEG72976.1|
gi|334193255|gb|AEG72983.1|
gi|334193259|gb|AEG72987.1|
gi|334193112|gb|AEG72840.1|
gi|334193241|gb|AEG72969.1|
gi|334193243|gb|AEG72971.1|
gi|334193247|gb|AEG72975.1|
gi|334193185|gb|AEG72913.1|
gi|334193244|gb|AEG72972.1|
gi|334193161|gb|AEG72889.1|
gi|334193180|gb|AEG72908.1|
gi|334193158|gb|AEG72886.1|
gi|334193239|gb|AEG72967.1|
gi|334192961|gb|AEG72689.1|
gi|334192971|gb|AEG72699.1|
gi|334192984|gb|AEG72712.1|
gi|334192963|gb|AEG72691.1|
gi|334192965|gb|AEG72693.1|
gi|334193179|gb|AEG72907.1|

Family 3

---

gi|334193643|gb|AEG73371.1|
gi|334193636|gb|AEG73364.1|
gi|334193597|gb|AEG73325.1|
gi|334193633|gb|AEG73361.1|
gi|334193575|gb|AEG73303.1|
gi|334193609|gb|AEG73337.1|
gi|334193650|gb|AEG73378.1|
gi|334193674|gb|AEG73402.1|
gi|334193172|gb|AEG72900.1|
gi|334193653|gb|AEG73381.1|

gi|334193107|gb|AEG72835.1|
gi|334193599|gb|AEG73327.1|
gi|334193612|gb|AEG73340.1|
gi|334193637|gb|AEG73365.1|
gi|334193638|gb|AEG73366.1|
gi|334193641|gb|AEG73369.1|
gi|334193645|gb|AEG73373.1|
gi|334193647|gb|AEG73375.1|
gi|334193656|gb|AEG73384.1|
gi|334193640|gb|AEG73368.1|
gi|334193573|gb|AEG73301.1|
gi|334193598|gb|AEG73326.1|
gi|334193630|gb|AEG73358.1|
gi|334193649|gb|AEG73377.1|
gi|334193178|gb|AEG72906.1|
gi|334193652|gb|AEG73380.1|

Family 4

gi|334193315|gb|AEG73043.1|
gi|334193333|gb|AEG73061.1|
gi|334193317|gb|AEG73045.1|
gi|334193328|gb|AEG73056.1|
gi|334193332|gb|AEG73060.1|
gi|334193320|gb|AEG73048.1|
gi|334193324|gb|AEG73052.1|
gi|334193327|gb|AEG73055.1|
gi|334193321|gb|AEG73049.1|
gi|334193311|gb|AEG73039.1|
gi|334193326|gb|AEG73054.1|
gi|334193312|gb|AEG73040.1|
gi|334193329|gb|AEG73057.1|
gi|334193330|gb|AEG73058.1|
gi|334193316|gb|AEG73044.1|
gi|334193331|gb|AEG73059.1|
gi|334193322|gb|AEG73050.1|
gi|334193318|gb|AEG73046.1|

Family 5

gi|334193807|gb|AEG73535.1|
gi|334193569|gb|AEG73297.1|
gi|334193591|gb|AEG73319.1|
gi|334193564|gb|AEG73292.1|
gi|334193800|gb|AEG73528.1|
gi|334193799|gb|AEG73527.1|

gi|334193567|gb|AEG73295.1|
gi|334193589|gb|AEG73317.1|
gi|334193859|gb|AEG73587.1|
gi|334193565|gb|AEG73293.1|
gi|334193593|gb|AEG73321.1|
gi|334193804|gb|AEG73532.1|
gi|334193571|gb|AEG73299.1|
gi|334193108|gb|AEG72836.1|
gi|334193101|gb|AEG72829.1|
gi|334193110|gb|AEG72838.1|
gi|334193570|gb|AEG73298.1|

Family 6

---

gi|334193287|gb|AEG73015.1|
gi|334193443|gb|AEG73171.1|
gi|334193801|gb|AEG73529.1|
gi|334193803|gb|AEG73531.1|
gi|334193806|gb|AEG73534.1|
gi|334193590|gb|AEG73318.1|
gi|334193566|gb|AEG73294.1|
gi|334193595|gb|AEG73323.1|
gi|334193592|gb|AEG73320.1|
gi|334193100|gb|AEG72828.1|
gi|334193102|gb|AEG72830.1|

Family 7

---

gi|334193209|gb|AEG72937.1|
gi|334193277|gb|AEG73005.1|
gi|334193215|gb|AEG72943.1|
gi|334193146|gb|AEG72874.1|
gi|334193211|gb|AEG72939.1|
gi|334193137|gb|AEG72865.1|
gi|334193142|gb|AEG72870.1|
gi|334193145|gb|AEG72873.1|
gi|334193139|gb|AEG72867.1|
gi|334193208|gb|AEG72936.1|

Family 8

---

gi|334193952|gb|AEG73680.1|
gi|334193891|gb|AEG73619.1|
gi|334193831|gb|AEG73559.1|

gi|334193830|gb|AEG73558.1|
gi|334192762|gb|AEG72490.1|
gi|334192775|gb|AEG72503.1|
gi|334192776|gb|AEG72504.1|
gi|334192770|gb|AEG72498.1|

Family 9

gi|334193207|gb|AEG72935.1|
gi|334193214|gb|AEG72942.1|
gi|334193276|gb|AEG73004.1|
gi|334193141|gb|AEG72869.1|
gi|334193709|gb|AEG73437.1|
gi|334193272|gb|AEG73000.1|
gi|334193364|gb|AEG73092.1|

Family 10

gi|334193130|gb|AEG72858.1|
gi|334193197|gb|AEG72925.1|
gi|334193028|gb|AEG72756.1|
gi|334193282|gb|AEG73010.1|
gi|334193148|gb|AEG72876.1|
gi|334193059|gb|AEG72787.1|

Family 11

gi|334192761|gb|AEG72489.1|
gi|334192774|gb|AEG72502.1|
gi|334192767|gb|AEG72495.1|
gi|334192773|gb|AEG72501.1|
gi|334192771|gb|AEG72499.1|

Family 12

gi|334193906|gb|AEG73634.1|
gi|334193925|gb|AEG73653.1|
gi|334193893|gb|AEG73621.1|
gi|334193861|gb|AEG73589.1|

Family 13

gi|334193366|gb|AEG73094.1|
gi|334193342|gb|AEG73070.1|
gi|334193341|gb|AEG73069.1|
gi|334193365|gb|AEG73093.1|

Family 14

---

gi|334193089|gb|AEG72817.1|
gi|334193360|gb|AEG73088.1|
gi|334193086|gb|AEG72814.1|
gi|334193543|gb|AEG73271.1|

Family 15

---

gi|334193453|gb|AEG73181.1|
gi|334193399|gb|AEG73127.1|
gi|334193494|gb|AEG73222.1|
gi|334193448|gb|AEG73176.1|

Families of 3

---

gi|334193295|gb|AEG73023.1|
gi|334193257|gb|AEG72985.1|
gi|334193294|gb|AEG73022.1|

gi|334192646|gb|AEG72374.1|
gi|334192648|gb|AEG72376.1|
gi|334192647|gb|AEG72375.1|

gi|334193363|gb|AEG73091.1|
gi|334193271|gb|AEG72999.1|
gi|334193339|gb|AEG73067.1|

gi|334193957|gb|AEG73685.1|
gi|334192734|gb|AEG72462.1|
gi|334192731|gb|AEG72459.1|

gi|334193274|gb|AEG73002.1|
gi|334193275|gb|AEG73003.1|

gi|334193279|gb|AEG73007.1|

gi|334193417|gb|AEG73145.1|
gi|334193418|gb|AEG73146.1|
gi|334193416|gb|AEG73144.1|

gi|334192952|gb|AEG72680.1|
gi|334193083|gb|AEG72811.1|
gi|334192889|gb|AEG72617.1|

gi|334193280|gb|AEG73008.1|
gi|334193147|gb|AEG72875.1|
gi|334193216|gb|AEG72944.1|

gi|334193938|gb|AEG73666.1|
gi|334193937|gb|AEG73665.1|
gi|334193892|gb|AEG73620.1|

gi|334192929|gb|AEG72657.1|
gi|334193034|gb|AEG72762.1|
gi|334192937|gb|AEG72665.1|

Families of 2

gi|334192821|gb|AEG72549.1|
gi|334192822|gb|AEG72550.1|

gi|334193301|gb|AEG73029.1|
gi|334193864|gb|AEG73592.1|

gi|334193936|gb|AEG73664.1|
gi|334193935|gb|AEG73663.1|

gi|334193334|gb|AEG73062.1|
gi|334193325|gb|AEG73053.1|

gi|334193695|gb|AEG73423.1|
gi|334193608|gb|AEG73336.1|

gi|334192698|gb|AEG72426.1|

gi|334192697|gb|AEG72425.1|

gi|334193687|gb|AEG73415.1|
gi|334193684|gb|AEG73412.1|

gi|334193985|gb|AEG73713.1|
gi|334193983|gb|AEG73711.1|

gi|334193814|gb|AEG73542.1|
gi|334193817|gb|AEG73545.1|

gi|334192747|gb|AEG72475.1|
gi|334192811|gb|AEG72539.1|

gi|334193361|gb|AEG73089.1|
gi|334193338|gb|AEG73066.1|

gi|334192789|gb|AEG72517.1|
gi|334193895|gb|AEG73623.1|

gi|334192682|gb|AEG72410.1|
gi|334192683|gb|AEG72411.1|

gi|334193499|gb|AEG73227.1|
gi|334193495|gb|AEG73223.1|

gi|334192991|gb|AEG72719.1|
gi|334192989|gb|AEG72717.1|

gi|334193149|gb|AEG72877.1|
gi|334193283|gb|AEG73011.1|

gi|334193715|gb|AEG73443.1|
gi|334193710|gb|AEG73438.1|

gi|334193038|gb|AEG72766.1|
gi|334193039|gb|AEG72767.1|

gi|334193097|gb|AEG72825.1|
gi|334193168|gb|AEG72896.1|

gi|334193018|gb|AEG72746.1|
gi|334192921|gb|AEG72649.1|
